# Improved methods for estimating abundance and related demographic parameters from mark‐resight data

**DOI:** 10.1111/biom.13058

**Published:** 2019-04-25

**Authors:** Brett T. McClintock, Gary C. White, Moira A. Pryde

**Affiliations:** ^1^ Marine Mammal Laboratory, Alaska Fisheries Science Center NOAA National Marine Fisheries Service Seattle Washington; ^2^ Department of Fish, Wildlife, and Conservation Biology Colorado State University Fort Collins Colorado; ^3^ New Zealand Department of Conservation Wellington New Zealand

**Keywords:** capture‐recapture, capture resight, population size, Program NOREMARK, survival

## Abstract

Over the past decade, there has been much methodological development for the estimation of abundance and related demographic parameters using mark‐resight data. Often viewed as a less‐invasive and less‐expensive alternative to conventional mark recapture, mark‐resight methods jointly model marked individual encounters and counts of unmarked individuals, and recent extensions accommodate common challenges associated with imperfect detection. When these challenges include both individual detection heterogeneity and an unknown marked sample size, we demonstrate several deficiencies associated with the most widely used mark‐resight models currently implemented in the popular capture‐recapture freeware Program MARK. We propose a composite likelihood solution based on a zero‐inflated Poisson log‐normal model and find the performance of this new estimator to be superior in terms of bias and confidence interval coverage. Under Pollock's robust design, we also extend the models to accommodate individual‐level random effects across sampling occasions as a potentially more realistic alternative to models that assume independence. As a motivating example, we revisit a previous analysis of mark‐resight data for the New Zealand Robin (*Petroica australis*) and compare inferences from the proposed estimators. For the all‐too‐common situation where encounter rates are low, individual detection heterogeneity is non‐negligible, and the number of marked individuals is unknown, we recommend practitioners use the zero‐inflated Poisson log‐normal mark‐resight estimator as now implemented in Program MARK.

## 1 INTRODUCTION

Mark‐resight methods are commonly used to estimate abundance and related demographic parameters for animal populations (eg, White and Shenk, 2001; McClintock and White, 2012). They are widely applicable across diverse taxa, including terrestrial mammals (eg, Keech *et al.*, 2011), marine mammals (eg, Ryan *et al.*, 2011), and birds (eg, Lyons *et al.*, 2016). Mark‐resight methods are most often utilized when some segment of the population is marked, and non‐invasive sighting data can be simultaneously collected for both marked and unmarked individuals. Non‐invasive sighting data can include those arising from ground (eg, McClintock and White, 2007), aerial (eg, Keech *et al.*, 2011), and camera (eg, Alonso *et al.*, 2015) surveys. Because mark‐resight does not require repeated physical capture of individuals, the main advantage of this approach is that costs associated with marking and recapturing can be minimized. In many circumstances, it can therefore be a less‐invasive and less‐expensive alternative to traditional mark recapture for population monitoring. Relative to capture recapture, mark‐resight abundance estimators also tend to be more robust to individual heterogeneity in detection probability because marked individuals are selected independent of the sighting process (ie, “all‐zero” detection histories are observed).

Mark‐resight estimators have seen much development over the past decade (McClintock and White, 2012; Matechou *et al.*, 2013; Alonso *et al.*, 2015; Lyons *et al.*, 2016), and the models that are currently implemented in the freeware Program MARK (White and Burnham, 1999) are widely used by practitioners. Because it does not require knowledge of the exact number of marked individuals in the population, one of the most popular mark‐resight models for estimating abundance and survival parameters is the zero‐truncated Poisson log‐normal estimator (ztPNE; McClintock *et al.*, 2009; McClintock & White, 2009). Here, we identify several potential deficiencies of the ztPNE currently implemented in Program MARK. Among them are its failure to utilize valuable information about the number of marks ever released into the population and, under Pollock's robust design, its conditioning on first *sighting* instead of first *encounter* (eg., capture). The ztPNE in Program MARK also relies on an ad‐hoc adjustment term to account for marks that are sighted but not identified to individual identity (see McClintock *et al.*, 2014). Lastly, while the ztPNE implemented in Program MARK can include individual‐level random effects in detection probability, these individual heterogeneity effects are (perhaps unrealistically) assumed to be independent across primary sampling periods.

As a solution to these potential deficiencies of ztPNE, we propose the zero‐inflated Poisson log‐normal estimator (ziPNE). By allowing for zero inflation in the marked individual sighting data, ziPNE can incorporate all marked individuals ever released into the population and thus does not need to condition on first sighting. We also extend the likelihoods for these models to accommodate unidentified marks and individual‐level random effects across primary sampling occasions. After describing ziPNE in the next section, we compare the performance of ziPNE relative to ztPNE under a broad range of simulated sampling scenarios. We then revisit the New Zealand Robin (*Petroica australis*) analysis of McClintock and White (2009) and compare inferences from the various estimators.

## 2 METHODS

There are several different study designs for the mark‐resight models in Program MARK (McClintock and White, 2012). The mark‐resight scenario under consideration consists of T sampling occasions where sightings of both marked and unmarked individuals are recorded. The observable population size $\left(N_{t}\right)$ is defined as the number of individuals that are available to be observed (eg, alive and within the study area) during sighting survey t=1,…,T. The population is assumed to be geographically and demographically closed within each survey, but not between surveys. To account for temporary emigration off the study area (or any other process that causes a segment of the population to be temporarily unobservable), we define the unobservable population size as the number of individuals that were alive during survey t but had temporarily emigrated off the study area between times t−1 and t.

During each survey, there are $n_{t}\in \left\{0,\ldots ,M_{t}\right\}$ marked individuals and $U_{t}=N_{t}-n_{t}$ unmarked individuals in the observable population, where $M_{t}=\sum_{k=1}^{t}R_{k}$ is the total number of marked individuals ever released into the population prior to survey t, and $R_{t}$ is the number of (unmarked) individuals that were newly marked between times t−1 and t. The mark‐resight data therefore consist of the number of resightings for each marked individual ($x_{s,t}\in \left\{0,1,2,\;\ldots \right\}$ for $s\in M_{t}$) and the total number of unmarked individual sightings $\left(u_{t}=\sum_{s\notin M_{t}}x_{s,t}\right)$, where $M_{t}$ is the set of $M_{t}$ marked individuals ever released into the population prior to survey t. In the common circumstance where the identity of an individual detected as marked cannot always be determined (McClintock *et al.*, 2014), then the observed data for the marked segment of the population consist of $y_{s,t}=x_{s,t}-\epsilon_{s,t}$ for $s\in M_{t}$ and the total number of marked sightings that were not individually identified $\left(e_{t}=\sum_{s\in M_{t}}\epsilon_{s,t}\right)$. The number of marks alive and within the study area can sometimes be reasonably assumed to be known exactly (eg, if all $n_{t}$ marks are introduced immediately prior to sighting survey t), but more commonly $n_{t}$ is unknown due to attrition of marks over time from mortality and permanent emigration.

When $n_{t}$ is unknown for at least one sampling occasion, the zero‐truncated Poisson log‐normal estimator (ztPNE, McClintock *et al.*, 2009; McClintock and White, 2009) can be used to estimate abundance $\left(N_{t}\right)$, apparent survival from time t to time $t+1\;\left(\phi_{t}\right)$, the transition probability from an observable state (eg. in the study area) at time t to an unobservable state (eg, temporarily off the study area) at time $t+1\;\left(\gamma_{t}\prime \prime \right)$, and the transition probability from an unobservable state at time t to an unobservable state at time $t+1\;\left(\gamma_{t}\prime \right)$. However, the ztPNE does not capitalize on all of the available information afforded by this mark‐resight study design. Notably, the model does not incorporate $M_{t}$ into estimation of detection probability parameters, and, under Pollock's robust design (Kendall *et al.*, 1997; McClintock and White, 2009), it conditions on the first *sighting* of each marked individual (instead of first encounter; eg, capture) for estimating the open population parameters (ϕ,γ′, and γ′′).

To make better use of the available information, we propose the zero‐inflated Poisson log‐normal estimator (ziPNE). The assumptions of ziPNE are identical to the standard mark‐resight assumptions of ztPNE (McClintock *et al.*, 2009; McClintock and White, 2009). Unmodeled individual sighting heterogeneity is well known to induce bias in abundance estimators, and we develop two ziPNE formulations based on whether or not individual‐level effects for sighting rates are assumed independent (“within” heterogeneity *sensu* McClintock *et al.*, 2009) or not (“across” heterogeneity *sensu* Gimenez and Choquet, 2010) between sampling occasions.

Assuming the latent $x_{s,t}$ sightings follow a Poisson log‐normal distribution, we have $x_{s,t}|\lambda_{s,t}\;\sim \;\text{Poisson}\left(\lambda_{s,t}\right),\;y_{s,t}|\lambda_{s,t},r_{t}\;\sim \;\text{Poisson}\left(\lambda_{s,t}r_{t}\right)$, and $\epsilon_{s,t}|\lambda_{s,t},r_{t}\;\sim \;\text{Poisson}\left(\lambda_{s,t}\left(1-r_{t}\right)\right)$, where $\lambda_{s,t}=\text{exp}\left(\alpha_{t}+\sigma_{t}z_{s,t}\right)$ and $r_{t}$ is the conditional probability that a mark is individually identified (given detected as marked). The individual sighting rate $\left(\lambda_{s,t}\right)$ includes a (log‐scale) mean $\left(\alpha_{t}\right)$, standard deviation $\left(\sigma_{t}\right)$, and latent individual‐level effect $z_{s,t}\;\sim \;N\left(0,1\right)$. When the latent individual‐level effects include time dependence, we refer to this as the “within” heterogeneity model. For an “across” heterogeneity model, we simply remove time dependence from the individual effects: $\lambda_{s,t}=\text{exp}\left(\alpha_{t}+\sigma_{t}z_{s}\right)$, where $z_{s}\;\sim \;N\left(0,1\right)$.

Under this scenario, likelihood‐based estimation of $N_{t}$ and related demographic parameters is challenging for several reasons. The individual‐level effects clearly induce dependencies between $Y=\left(y_{s,t}\right)$ and $e=\left(e_{1},\ldots ,e_{T}\right)$, and, for the “across” heterogeneity models, within Y,e, and $u=\left(u_{1},\ldots ,u_{T}\right)$. A further complication for the “across” heterogeneity models is that when $R_{t}>0$ for t∈{2,…,T}, then $y_{s,t:T},\;e_{t:T}$, and $u_{1:t-1}$ may not be independent for $s\notin M_{t-1}$. One possible solution is to formulate a complete data likelihood and use data augmentation (eg, McClintock *et al.*, 2014; Rankin *et al.*, 2016), but such approaches can be time‐consuming and difficult to fit (see Web Appendix A). We instead adopt a practical perspective and focus our efforts on a composite likelihood (eg, Varin *et al.*, 2011) that is amenable to maximum likelihood estimation and implementation in Program MARK. Web Table B1 includes definitions for all parameters, latent variables, and random variables in our composite likelihood.

To make the problem tractable, we propose to ignore any covariance between Y,e, and u and simply integrate over the latent Z for each of three components of a composite observed data likelihood as if they were independent:

(1) $$\begin{array}{ccc} \left[Y,u,e|U,\alpha ,\sigma ,r,w,g,\phi ,\gamma \prime ,\gamma \prime \prime \right] & = & \prod_{s=1}^{M_{T}}\int_{z_{s}}\left[y_{s}|z_{s},\alpha ,\sigma ,r,w,g,\phi ,\gamma \prime ,\gamma \prime \prime \right]\left[z_{s}\right]\\ & \times & \int_{Z}\left[e|Z,\alpha ,\sigma ,r\right]\left[Z\right]\\ & \times & \int_{Z}\left[u|Z,U,\alpha ,\sigma \right]\left[Z\right], \end{array}$$

where bracket notation [data∣parameter] denotes a probability density (or mass) function. We can account for zero inflation in $y_{s}$ by assuming marked individuals are in one of three states at time t (1 = dead or permanent emigrant; 2 = alive, not a permanent emigrant, off the study area; 3 = alive, not a permanent emigrant, on the study area). Let $w_{t}=\left[1-w_{t},w_{t}\left(1-g_{t}\right),w_{t}g_{t}\right]$ be a row vector of initial state probabilities, where $w_{t}$ is the probability that a randomly selected individual from the $R_{t}$ new marks introduced between sighting occasion t−1 and t was alive and not a permanent emigrant during sighting occasion t, and $g_{t}$ is the conditional probability that this individual was within the study area (given alive and not a permanent emigrant). Let $P_{s,t}$ be a 3×3 matrix where the diagonal elements denote the probability mass function of $y_{s,t}$ conditional on the state of individual s at time t, and let $G_{t}$ be a 3×3 state transition probability matrix with rows corresponding to the state at time t and the columns corresponding to the state at time t+1. We then have

$$\begin{array}{c} \left[y_{s}|z_{s},\alpha ,\sigma ,r,w,g,\phi ,\gamma \prime ,\gamma \prime \prime \right]=w_{b_{s}}P_{s,b_{s}}\left[\prod_{i=b_{s}}^{T-1}G_{i}P_{s,i+1}\right]1_{3} \end{array}$$

$$P_{s,t}=\left[\begin{array}{ccc} 1-I\left(y_{s,t}>0\right) & 0 & 0\\ 0 & 1-I\left(y_{s,t}>0\right) & 0\\ 0 & 0 & p_{s,t} \end{array}\right],$$

$$G_{t}=\left[\begin{array}{ccc} 1 & 0 & 0\\ 1-\phi_{t} & \phi_{t}\gamma_{t}\prime & \phi_{t}\left(1-\gamma_{t}\prime \right)\\ 1-\phi_{t} & \phi_{t}\gamma_{t}\prime \prime & \phi_{t}\left(1-\gamma_{t}\prime \prime \right) \end{array}\right],$$

where $b_{s}\in \left\{1,\ldots ,T\right\}$ is the first sighting occasion for which individual s was marked (and thus potentially available for resighting), I() is the indicator function, $1_{3}={\left[1,1,1\right]}^{T}$, and $p_{s,t}=\left[y_{s,t}|\lambda_{s,t},r_{t}\right]$. By allowing $y_{s,t}$ for the $R_{t}$ newly marked individuals to be zero inflated (with probability $1-w_{t}g_{t}$), we can include $M_{T}$ and condition on first encounter (instead of first sighting) in our composite likelihood.

For both the “within” and “across” models, we wish to integrate over the latent variables $z_{s,t}$ and $z_{s}$, respectively, in Equation (1). While this is straightforward for $\left[y_{s}|z_{s},\alpha ,\sigma ,r,w,g,\phi ,\gamma \prime ,\gamma \prime \prime \right],\;\left[u|U,\alpha ,\sigma \right]=\int_{Z}\left[u|Z,U,\alpha ,\sigma \right]\left[Z\right]$ and $\left[e|\alpha ,\sigma ,r\right]=\int_{Z}\left[e|Z,\alpha ,\sigma ,r\right]\left[Z\right]$ pose difficulties because sums of Poisson log‐normal random variables are not of standard form (eg, Poisson) and they involve multidimensional integrals that are not amenable to numerical approximation. Similar to ztPNE, we rely on a left‐truncated normal distribution for [u∣U,α,σ] based on the expected value $\lambda_{t}=E_{z}\left(x_{s,t}|\lambda_{s,t}\right)=\int E\left(x_{s,t}|\lambda_{s,t}\right)\left[z\right]dz=\int \text{exp}\left(\alpha_{t}+\sigma_{t}z\right)\left[z\right]dz=\text{exp}\left(\alpha_{t}+\sigma_{t}^{2}∕2\right)$ and variance $\eta_{t}={\text{var}}_{z}\left(x_{s,t}|\lambda_{s,t}\right)=E_{z}\left(\text{var}\left(x_{s,t}|\lambda_{s,t}\right)\right)+{\text{var}}_{z}\left(E\left(x_{s,t}|\lambda_{s,t}\right)\right)=\lambda_{t}+\text{exp}\left(2\alpha_{t}\right)\left(\text{exp}\left(2\sigma_{t}^{2}\right)-\text{exp}\left(\sigma_{t}^{2}\right)\right)$ of a Poisson log‐normal random variable after integrating over the individual‐level effects:

$$\left[u|U,\alpha ,\sigma \right]=\prod_{t=1}^{T}\left[u_{t}^{*}|U_{t},\alpha_{t},\sigma_{t}\right]$$

where $u_{t}^{*}|U_{t},\alpha_{t},\sigma_{t}\sim {\mathrm{TN}}_{\left(0,\infty \right)}\left(U_{t}\lambda_{t},U_{t}\eta_{t}\right),\;u_{t}^{*}=u_{t}$ is a non‐negative continuous random variable, and ${\mathrm{TN}}_{\left(a,b\right)}$ represents a normal distribution truncated at a and b. Note that for the “across” heterogeneity model, this composite likelihood also ignores the covariance induced in u by the latent individual‐level effects.

We employ a similar left‐truncated normal distribution for [e∣α,σ,r]:

(2) $$\left[e|\alpha ,\sigma ,r\right]=\prod_{t=1}^{T}\left[e_{t}^{*}|\alpha_{t},\sigma_{t},r_{t}\right]$$

where $e_{t}^{*}|\alpha_{t},\sigma_{t},r_{t}\sim {\mathrm{TN}}_{\left(0,\infty \right)}(n_{t}\lambda_{t}\left(1-r_{t}\right),n_{t}\kappa_{t}),\;\kappa_{t},\lambda_{t}\left(1-r_{t}\right)+\text{exp}\left(2\alpha_{t}\right){\left(1-r_{t}\right)}^{2}\left(\text{exp}\left(2\sigma_{t}^{2}\right)-\text{exp}\left(\sigma_{t}^{2}\right)\right)$, and $e_{t}^{*}=e_{t}$ is a non‐negative continuous random variable. While $n_{1}$ can sometimes be reasonably assumed to be known exactly (ie, $n_{1}=M_{1}$ and $w_{1}=g_{1}=1$), for all occasions where the number of marks alive and within the study area is not known exactly, we replace $n_{t}$ in Equation (2) with a Horvitz‐Thompson‐type estimator: ${\hat{n}}_{t}=\sum_{s\in M_{t}}I\left(y_{s,t}>0\right)p_{t}^{-1}$, where $p_{t}=1-\int \text{exp}\left(-\text{exp}\left(\alpha_{t}+\sigma_{t}z\right)r_{t}\right)\left[z\right]dz$ is the probability of a marked individual being resighted at least once.

We therefore have the following composite likelihood for the “within” heterogeneity ziPNE:

(3) $$\begin{array}{ccc} \left[Y,u,e|U,\alpha ,\sigma ,r,w,g,\phi ,\gamma \prime ,\gamma \prime \prime \right] & = & \prod_{s=1}^{M_{T}}\left[y_{s}|\alpha ,\sigma ,r,w,g,\phi ,\gamma \prime ,\gamma \prime \prime \right]\\ & \times & \prod_{t=1}^{T}\left[e_{t}^{*}|\alpha_{t},\sigma_{t},r_{t}\right]\left[u_{t}^{*}|U_{t},\alpha_{t},\sigma_{t}\right], \end{array}$$

where $p_{s,t}=\int \left[y_{s,t}|\lambda_{s,t},r_{t}\right]\left[z_{s,t}\right]dz_{s,t}$ and $\lambda_{s,t}=\text{exp}\left(\alpha_{t}+\sigma_{t}z_{s,t}\right)$. For the “across” heterogeneity ziPNE:

(4) $$\begin{array}{ccc} \left[Y,u,e|U,\alpha ,\sigma ,r,w,g,\phi ,\gamma \prime ,\gamma \prime \prime \right] & = & \prod_{s=1}^{M_{T}}\int \left[y_{s}|z_{s},\alpha ,\sigma ,r,w,g,\phi ,\gamma \prime ,\gamma \prime \prime \right]\left[z_{s}\right]dz_{s}\\ & \times & \prod_{t=1}^{T}\left[e_{t}^{*}|\alpha_{t},\sigma_{t},r_{t}\right]\left[u_{t}^{*}|U_{t},\alpha_{t},\sigma_{t}\right], \end{array}$$

where $p_{s,t}=\left[y_{s,t}|\lambda_{s,t},r_{t}\right]$ and $\lambda_{s,t}=\text{exp}\left(\alpha_{t}+\sigma_{t}z_{s}\right)$. For maximum likelihood analysis, the integrals in Equations (3) and (4) can be easily approximated using Gauss‐Hermite quadrature:

$$p_{s,t}=\int \left[[,y_{s,t}|\lambda_{s,t},r_{t}\right]\left[z_{s,t}\right]dz_{s,t}\approx \frac{1}{\sqrt{\pi }}\sum_{l=1}^{L}f_{l}\left[[,y_{s,t}|\lambda_{l,t}^{*},r_{t},]\right],$$

and

$$\int \left[y_{s}|z_{s},\alpha ,\sigma ,r,w,g,\phi ,\gamma \prime ,\gamma \prime \prime \right]\left[z_{s}\right]dz_{s}\approx \frac{1}{\sqrt{\pi }}\sum_{l=1}^{L}f_{l}\left\{w_{b_{s}}P_{l,b_{s}}^{*}\left[\prod_{i=b_{s}}^{T-1}G_{i}P_{l,i+1}^{*}\right]1_{3}\right\},$$

where

$$P_{l,t}^{*}=\left[\begin{array}{ccc} 1-I\left(y_{s,t}>0\right) & 0 & 0\\ 0 & 1-I\left(y_{s,t}>0\right) & 0\\ 0 & 0 & \left[y_{s,t}|\lambda_{l,t}^{*},r_{t}\right] \end{array}\right],$$

$y_{s,t}|\lambda_{l,t}^{*},r_{t}\;\sim \;\text{Poisson}\left(\lambda_{l,t}^{*}r_{t}\right),\;\lambda_{l,t}^{*}=\text{exp}\left(\alpha_{t}+\sqrt{2}\sigma_{t}v_{l}\right)$, and $\left(f_{l},v_{l}\right)$ are the corresponding weights and nodes, respectively, for quadrature points l=1,…,L (Abramowitz and Stegun, 1964). When $n_{t}$ is unknown, we have $p_{t}\approx 1-\frac{1}{\sqrt{\pi }}\sum_{l=1}^{L}f_{l}\;\text{exp}\left(-\lambda_{l,t}^{*}r_{t}\right)$.

The size of the observable population (eg, alive and within the study area) during sighting survey $t\;\left(N_{t}=U_{t}+n_{t}\right)$ can be derived from the estimated parameters after fitting Equations (3) or (4) using maximum likelihood methods. As with all derived parameters in Program MARK, standard errors for $N_{t}$ are estimated using the delta method and finite‐difference approximations of the first derivative. Program MARK also facilitates modeling any of the parameters as a function of covariates using link functions (see Section 4). We note that in a fully time‐dependent model for $w_{t}$ and $g_{t}\;\left(t=1,\ldots ,T\right)$, only the product $w_{T}g_{T}$ is estimable. When T=1, only the product $w_{1}g_{1}$ is estimable. We also note that because the “within” and “across” heterogeneity models use the same data, they can be compared using model selection criteria such as Akaike's Information Criterion (AIC; Burnham and Anderson, 2002).

## 3 SIMULATION STUDY

Using data generated from the exact model, we performed a series of simulation experiments to evaluate the performance of our composite ziPNE relative to the ztPNE as implemented in Program MARK (McClintock and White, 2012. Each simulated scenario included $T=7,\phi_{t}=0.95,\;\gamma_{t}\prime \prime =\gamma_{t}\prime =0.1,\;w_{t}=0.95,\;g_{t}=0.9,\;r_{t}\in \left\{0.5,0.9\right\},\;E\left(R_{1}\right)\in \left\{60,120\right\},\;E\left(U_{1}\right)\in \left\{300,600\right\},\;\lambda_{t}\in \left\{1.5,5,100\right\}$, and τ∈{0,0.5,1,5}, where ${\text{var}}_{z}\left(x_{s,t}|\lambda_{s,t}\right)=\lambda_{t}\left(1+\tau \right)$, such that $\alpha_{t}=2\log\left(\lambda_{t}\right)-\log\left(\lambda_{t}^{2}+\lambda_{t}\tau \right)∕2$ and $\sigma_{t}=\sqrt{\log\left(\lambda_{t}^{2}+\lambda_{t}\tau \right)-2\log\left(\lambda_{t}\right)}$ for t=1,…,T. For $R_{t}\;\left(t=2,\ldots ,T\right)$, some of the $U_{t-1}$ unmarked individuals became newly marked (with probability 0.02) in order to maintain a somewhat consistent marked population size over time. However, no additional unmarked individuals were introduced to the population after the first occasion, so $N_{t}$ is expected to steadily decline through time due to mortality and permanent emigration. Scenarios with $\lambda_{t}=100$ represent the “best case” scenario where sighting rates are very high and individuals that are alive and within the study area are virtually guaranteed to be sighted (i.e. $x_{s,t}>0$). Under this scenario, we would expect ziPNE and ztPNE to perform similarly. Scenarios with both $\lambda_{t}=1.5$ and τ=5 represent the pathological case where mean sighting rates are low and individual heterogeneity is high, whereby the vast majority of individuals are never expected to be sighted ($x_{s,t}=0$) but some individuals will be sighted many times ($x_{s,t}\gg 0$). In terms of α,σ, and N estimation, we would generally expect all estimators to perform less than nominally under this extreme scenario (particularly when $r_{t}=0.5$). While we would not expect ϕ estimators to be biased by extreme heterogeneity coupled with low mean sighting rates, we suspect it could also be deleterious to reliable estimation of γ′′ and γ′.

For each combination, we simulated data from both the “within” and “across” heterogeneity models, thus yielding 2×2×2×2×3×4=192 simulated scenarios. For each scenario, we fit the two models corresponding to the data‐generating heterogeneity model: 1) ziPNE within heterogeneity Equation (3) and ztPNE within heterogeneity; or 2) ziPNE across heterogeneity Equation (4) and ztPNE across heterogeneity. We investigated heterogeneity model mis‐mispecification in an additional series of simulation experiments (see Web Appendix E). We assumed that $n_{1}$ was known, $n_{t}\;\left(t=2,\ldots ,T\right)$ was unknown, and $M_{1}=n_{1}$. For scenarios with τ=0, we fixed $\sigma_{t}=0$ for each of the fitted models. For simplicity, we also fixed $w_{1}=g_{1}=1$ (because $n_{1}$ is known and $n_{1}=M_{1}$) and otherwise assumed no time dependence in parameters (eg, $\phi_{1}=\phi_{2}=\cdots =\phi_{T-1}$). All simulations used L=101 quadrature points for numerical integration. We evaluated estimator performance based on bias, 95% confidence interval coverage of the true parameter values, and root mean squared error (RMSE). Simulation results are presented using the nested loop plot of Rücker and Schwarzer (2014). Similar to a time series plot, nested loop plots serve to present a large number of simulation results by putting all scenarios into a lexicographical order and arranging them consecutively along the horizontal axis. The quantity of interest (eg, bias, coverage, and RMSE) is then plotted on the vertical axis.

**Figure 1:**
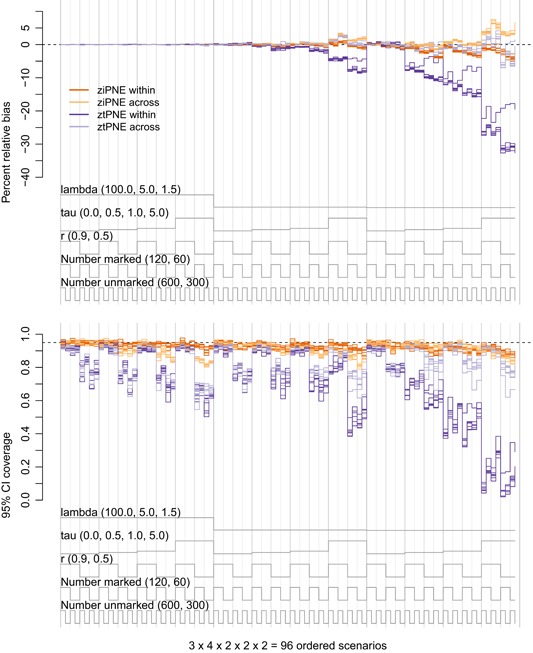
Nested loop plots of percent relative bias (top panel) and 95% confidence interval coverage (bottom panel) of abundance $\left(N_{t},t=1,\ldots ,7\right)$ estimators based on 192 simulated scenarios. Scenarios are ordered from outer to inner loops by $\lambda_{t}\in \left\{100,5,1.5\right\}$ (“lambda”; three levels, decreasing), τ∈{0,0.5,1,5} (“tau”; four levels, increasing), r∈{0.9,0.5} (two levels, decreasing), $E\left(R_{1}\right)\in \left\{120,60\right\}$ (“Number marked”; two levels, decreasing), and $E\left(U_{1}\right)\in \left\{600,300\right\}$ (“Number unmarked”; two levels, decreasing) such that the left‐ and right‐most scenarios are the best‐ and worst‐case sampling scenarios, respectively. Estimators include the zero‐inflated Poisson log‐normal estmator (*dark orange* = ziPNE within, *light orange* = ziPNE across) and the zero‐truncated Poisson log‐normal estimator (*dark purple* = ztPNE within, *light purple* = ztPNE across). Results are based on 400 simulated data sets for each scenario. This figure appears in color in the electronic version of this article, and color refers to that version

**Figure 2:**
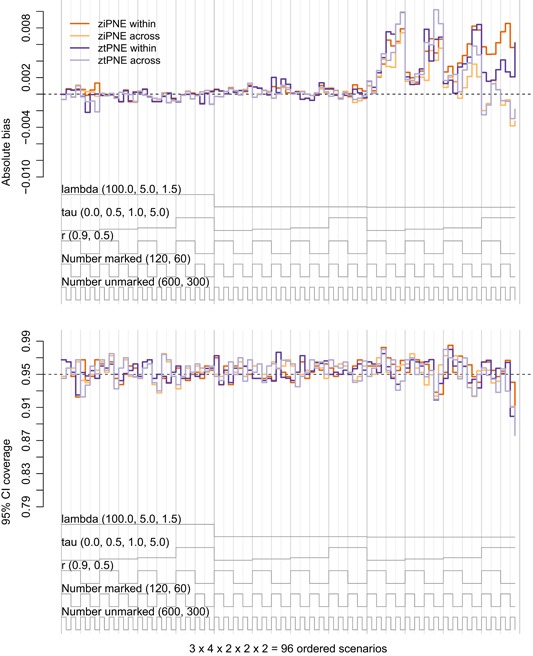
Nested loop plots of absolute bias (top panel) and 95% confidence interval coverage (bottom panel) of apparent survival (ϕ) estimators based on 192 simulated scenarios. Scenarios are ordered from outer to inner loops by $\lambda_{t}\in \left\{100,5,1.5\right\}$ (“lambda”; three levels, decreasing), τ∈{0,0.5,1,5} (“tau”; four levels, increasing), r∈{0.9,0.5} (two levels, decreasing), $E\left(R_{1}\right)\in \left\{120,60\right\}$ (“Number marked”; two levels, decreasing), and $E\left(U_{1}\right)\in \left\{600,300\right\}$ (“Number unmarked”; two levels, decreasing) such that the left‐ and right‐most scenarios are the best‐ and worst‐case sampling scenarios, respectively. Estimators include the zero‐inflated Poisson log‐normal estmator (*dark orange* = ziPNE within, *light orange* = ziPNE across) and the zero‐truncated Poisson log‐normal estimator (*dark purple* = ztPNE within, *light purple* = ztPNE across). Results are based on 400 simulated data sets for each scenario. This figure appears in color in the electronic version of this article, and color refers to that version

We found the “within” heterogeneity ziPNE to be far superior to ztPNE for the estimation of the closed population parameters (N,α, and σ), particularly as $\lambda_{t}$ decreased, τ increased, and r decreased. In nearly all of the scenarios, ziPNE tended to be less biased with higher 95% confidence interval coverage and lower RMSE (Figure 1, Web Figures C1–C4). Although it performed much better than ztPNE, ziPNE still exhibited an increase in bias and decrease in coverage under the pathological scenarios with low mean resighting rates and extreme heterogeneity. While little difference was found between ziPNE and ztPNE in terms of apparent survival (ϕ) estimation (Figure 2), ziPNE performed much better than ztPNE in the estimation of both γ′′ and γ′ (Web Figures C5‐C8).

We did not find such drastic differences in the performance of the “across” heterogeneity ziPNE relative to ztPNE, but ziPNE did tend to perform better in the estimation of the closed population parameters (Figure 1, Web Figures C2‐C4). While the “across” ztPNE tended to exhibit slightly less bias and lower RMSE for N in the most extreme scenarios, the “across” ziPNE tended to yield higher 95% confidence interval coverage (particularly when $r_{t}=0.5$; Figure 1, Web Figure C1). Nevertheless, coverage of N still tended to be less than nominal when τ>0 and $r_{t}=0.5$ for all $\lambda_{t}$. For the open parameters (ϕ,γ′′,γ′), we found that both ziPNE and ztPNE performed equally well in terms of bias and confidence interval coverage (Figure 2), but RMSE for ϕ and γ′ in particular tended to be larger for ztPNE (Web Figures C5‐C8).

## 4 EXAMPLE: NEW ZEALAND ROBIN

As an example application, we return to the New Zealand Robin (*P. australis*) data originally analyzed using ztPNE by McClintock and White (2009). This mark‐resight study was conducted by the New Zealand Department of Conservation on three 100 ha study areas (“Knobs Flat”, “Smithy”, and “Walker Creek”) in Fiordland National Park, New Zealand. Between September 2003 and August 2007, capture and banding of both juvenile and adult birds occurred continuously throughout the breeding season (August‐March) and intermittently prior to sighting surveys conducted in March (postbreeding) and August (prebreeding) of each year from 2005 to 2007. Because marked birds could have died or permanently emigrated during the extended capture period, the exact number of marked individuals in each population was unknown for every sighting occasion. For each occasion, the observable population for abundance estimation consisted of all juvenile and adult Robins within a study area. Sighting surveys did not begin in Smithy until August 2005, so there were T=6 sighting occasions for both Knobs Flat and Walker Creek, but only T=5 occasions for Smithy. Notably, ship rat (*Rattus rattus*) control was conducted on the Walker Creek study area from June 2006 to April 2007. Complete details of the study design and data can be found in McClintock and White (2009).

We performed a ziPNE analysis for comparison to the “within” ztPNE analysis of McClintock and White (2009) using Program MARK. While the original ztPNE analysis only included detection histories for $\sum_{s\in M_{T}}I\left(\sum_{t=1}^{T}y_{s,t}>0\right)=149$ marked birds that were sighted at least once between March 2005 and August 2007, the ziPNE analysis includes detection histories for all $M_{T}=439$ marked birds that were released into the populations prior to the August 2007 sighting occasion. Covariates examined for sighting rate and open population parameters included age class (*age*), sex, age class at time of capture (*agecap*), study area (*area*), time, breeding season (*season*), and rat control on Walker Creek. As in McClintock and White (2009), we also included an interaction between age, sex, and breeding season (age*sex*season) to investigate potential effects of reproduction on adult survival. For model selection, we used AIC adjusted for small sample sizes (AICc; Burnham and Anderson, 2002). Three covariate models for rat control on Walker Creek were examined: (1) an immediate and constant effect during the entire program (*rat*); (2) an immediate effect with no effect thereafter (*rat1*); and (3) no immediate effect with a delayed effect thereafter (*rat2*). We also investigated models under completely random emigration, γ′′=γ′ (Kendall *et al.*, 1997), and models ignoring individual heterogeneity (σ=0) or temporary emigration (γ′′=γ′=0).

Similar to McClintock and White (2009), we first investigated various models for the sighting rate parameters (α,σ, and r) under the most general (but identifiable) time‐ and group‐dependent structure for U and the open parameters (ϕ,γ′′, and γ′). We proceeded by selecting the model structure for α,σ, and r best supported by AICc and then using only this structure for investigating more parsimonious models for ϕ,γ′′, and γ′ (for an evaluation of a similar model selection procedure, see Doherty *et al.*, 2012). We then fit all combinations of the three best supported structures for each parameter based on each stage of this selection process. We assumed the same structure for w and ϕ and the same structure for g,γ′′, and γ′. However, because they pertain to the entire capture period beginning in September 2003, we also investigated $w_{1}$ and $g_{1}$ effects (*cap1*) for the Knobs Flat and Walker Creek study areas, and $w_{2}$ and $g_{2}$ effects (*cap2*) for the Smithy study area. Unlike the “within” ztPNE analysis of McClintock and White (2009), we investigated both “within” and “across” heterogeneity models, and we again note that, unlike the ziPNE proposed here, the ztPNE of McClintock and White (2009) does not include the r parameter and instead relies on an ad‐hoc adjustment term for unidentified marks. When fitting the models, we found the Markovian temporary emigration models including additive *age*, *agecap*, or *area* effects to have suspect convergence and thus excluded these from our model set. We based our comparisons on model‐averaged parameter estimates and unconditional variances calculated from AICc weights.

AICc overwhelmingly supported “across” heterogeneity models for ziPNE, with the minimum AICc model including: *agecap*, *sex*, *season*, and *area* effects on ϕ and w; *cap1* and *cap2* effects on w; *agecap* and *season* effects on random emigration (ie, γ′′=γ′) and g; *age*, *sex*, and *rat* effects on α; *sex* effects on σ; and *area* by *time* interaction effects on r. Model selection was somewhat similar for the “within” ztPNE model of McClintock and White (2009), but the ziPNE analysis exhibited considerably more model selection uncertainty (Web Table D1) and weak evidence of rat control effects on ϕ.

**Figure 3:**
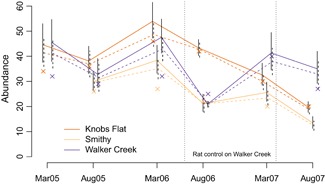
Mark‐resight abundance (N) estimates and 95% confidence intervals between March 2005 and August 2007 for three New Zealand Robin (*Petroica australis*) populations (*dark orange* = Knobs Flat, *light orange* = Smithy, and *dark purple* = Walker Creek) in Fiordland National Park, New Zealand. Estimates are AICc model averaged based on the zero‐inflated model (ziPNE; solid lines) and the zero‐truncated model (ztPNE; dashed lines). Crosses (×) indicate the minimum number known alive based on territory mapping that was independent of the mark‐resight methodology. Vertical dotted lines indicate a period of rat control on the Walker Creek study area. This figure appears in color in the electronic version of this article, and color refers to that version

**Figure 4:**
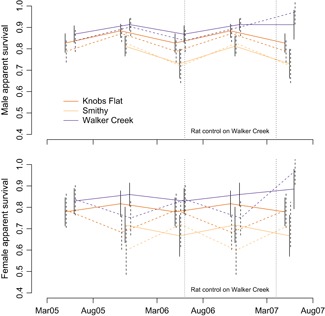
Model‐averaged four‐month apparent survival (ϕ) estimates (±SE) between March 2005 and August 2007 for three New Zealand Robin populations (*dark orange* = Knobs Flat, *light orange* = Smithy, and *dark purple* = Walker Creek) in Fiordland National Park, New Zealand. Estimates are for males (top panel) and females (bottom panel) that were first captured as adults based on the zero‐inflated model (ziPNE; solid lines) and the zero‐truncated model (ztPNE; dashed lines). The longer intervals between August and March encompass the breeding season. Vertical dotted lines indicate a period of rat control on the Walker Creek study area. This figure appears in color in the electronic version of this article, and color refers to that version

For ziPNE, model‐averaged mean sighting rates $\left(\lambda_{t}\right)$ were the lowest in August 2007 (2.2, 95% CI: 1.9−2.5) and the highest on Walker Creek during rat control in March 2007 (3.2, 95% CI: 2.6−4). For the “across” ziPNE models, model‐averaged σ=0.52 (SE =0.18) for females, and σ=0.28 (SE =0.07) for males. Individual mark identification probabilities (r) varied by study area and time but were generally high, with model‐averaged estimates ranging from 0.81 (SE =0.07) to 1.00 (SE =0.00). Point and variance estimates for $N_{t}$ tended to be larger for ziPNE, and, except for Walker Creek in August 2006, all estimated 95% confidence intervals for $N_{t}$ were greater than the minimum number known alive based on territory mapping that was independent of the mark‐resight methodology (Figure 3). As with the original ztPNE analysis, the ziPNE analysis found evidence of higher apparent survival probabilities for individuals that were first captured as adults (Figure 4) when compared to individuals that were first captured as juveniles (Web Figure D1), but ziPNE exhibited less seasonal variation in adult female apparent survival relative to ztPNE. Temporary emigration parameter (γ′′ and γ′) point estimates were similar between ziPNE and ztPNE, but ziPNE was generally more precise (Web Figures D2‐D4). Estimates for w after longer capture intervals tended to be the lowest, particularly for individuals that were first captured as juveniles. Estimates for g were generally high and tended to be somewhat higher for juveniles (Web Figures D5–D6).

## 5 DISCUSSION

We have proposed a new zero‐inflated version of the Poisson log‐normal mark‐resight estimator in Program MARK. Using simulation, we demonstrated ziPNE has superior performance relative to the zero‐truncated model of McClintock and White (2009). By accounting for zero inflation in the marked individual sighting data, ziPNE is able to utilize valuable information about detection rates provided by marked individuals that were released but never subsequently sighted. This not only makes ziPNE more robust to individual heterogeneity for reliable estimation of population size but also allows the likelihood for the open population parameters (ϕ,γ′′,γ′) to condition on first encounter (instead of first sighting). ziPNE thus makes better use of available data and, unlike ztPNE, allows $\gamma \prime_{1}$ to be estimated under Pollock's robust design.

In terms of abundance estimation, previous simulation studies also found the “within” heterogenity zero‐truncated model of McClintock and White (2009) to perform increasingly poorly with low sighting rates, high levels of individual heterogenity, and low‐marked individual identification probabilities (McClintock *et al.*, 2009; McClintock and White, 2009; McClintock *et al.*, 2014). Our extensive simulations included a broader range of more extreme scenarios, and the new ziPNE performed near nominally and better than ztPNE in almost every scenario examined. However, the average coverage of N could still be as low as 81% for the “across” ziPNE when high levels of individual heterogeneity were combined with low mark identification probabilities. While we have not encountered actual mark‐resight data as information poor as some of the scenarios included in our simulations, these pathological sampling scenarios provide valuable insights about the limitations of our composite likelihoods. Practitioners should carefully consider study designs that increase sighting rates, decrease individual heterogeneity, and increase marked individual identification whenever possible.

Unless changes in individual behavior (or sighting survey design) effectively eliminates correlations in the marked individual sighting data, the “across” model may often be a more natural choice than the “within” model. Indeed, our New Zealand Robin example demonstrated overwhelming support for the “across” models and thus strong correlations in the individual sighting data across sighting surveys. It deserves note that both the “across” and “within” models tended to perform poorly under heterogeneity model mis‐specification (see Web Appendix E). Regardless of the model (ztPNE or ziPNE), it is therefore important to examine both “within” and “across” heterogeneity models. Fortunately, these models can be compared using standard model selection criteria such as AIC and Bayesian information criterion (BIC). In some cases, it may be desirable to simultaneously model both “within” and “across” heterogeneity; this extension remains an avenue for future research.

Our ziPNE is a composite likelihood that ignores some of the dependencies induced by individual heterogeneity among Y,e, and u in order to facilitate maximum likelihood estimation in Program MARK. While this composite likelihood proved robust under a broad range of simulation scenarios, an alternative (exact) solution can be implemented using a data‐augmented complete data likelihood in a Bayesian framework (see Web Appendix A). While relatively easy to code using standard software, poor mixing can cause such implementations to be time consuming and challenging to fit. Thus, while conceptually appealing, they can often be impractical for simulation studies or real‐world application (particularly for large T or N). To aid practitioners in determining whether or not the performance of ziPNE can be expected to meet their specific objectives under sampling scenarios not covered in our simulations, we provide R code for simulating data from the exact model and fitting ziPNE in the Supporting Information.

We recommend practitioners use ziPNE for mark‐resight analyses in Program MARK whenever the number of marks in the population is unknown due to attrition arising from mortality and permanent emigration. As demonstrated in our New Zealand Robin example, we also recommend previous ztPNE analyses be revisited with ziPNE where appropriate. Program MARK includes both individual heterogeneity model formulations (“within” and “across”), and has many additional analysis features, such as covariate modeling via link functions, model selection using AIC or BIC, multimodel inference, and plotting. MARK also provides the ability to simulate data under the mark‐resight models, a useful feature for designing studies. For advanced users, these mark‐resight models can also be implemented through the R package RMark (Laake, 2013) interface for Program MARK.

## Supporting information

Supplementary Information

Supplementary Information

Supplementary Information
