# Supplementary material for: Improved methods for estimating abundance and related demographic parameters from mark‐resight data
Source: Biometrics. 2019 Apr 25;75(3):799–809. doi: 10.1111/biom.13058 (PMC6850357; doi:10.1111/biom.13058)
Supplement: Supplementary file 2 — Supplementary Information [file BIOM-75-799-s002.pdf]

Supporting Information for "Improved methods for estimating abundance and related demographic parameters from mark-resight data" by Brett T. McClintock, Gary C. White, and Moira A. Pryde.

Web Appendix A: Complete data likelihood formulation

Conditional on the latent $\mathbf{Z}$, we can formulate a complete data likelihood for the sighting data as:

$$[\mathbf{Y}, \mathbf{u}, \mathbf{e} \mid \mathbf{Z}, \boldsymbol{\alpha}, \boldsymbol{\sigma}, \boldsymbol{\psi}, \mathbf{h}, \boldsymbol{\phi}, \boldsymbol{\gamma}', \boldsymbol{\gamma}''] = \sum_{\mathbf{X}} [\mathbf{X} \mid \mathbf{Z}, \boldsymbol{\alpha}, \boldsymbol{\sigma}, \boldsymbol{\psi}, \mathbf{h}, \boldsymbol{\phi}, \boldsymbol{\gamma}', \boldsymbol{\gamma}''] [\mathbf{Y}, \mathbf{u}, \mathbf{e} \mid \mathbf{X}] \quad (5)$$

where

$$[\mathbf{X} \mid \mathbf{Z}, \boldsymbol{\alpha}, \boldsymbol{\sigma}, \boldsymbol{\psi}, \mathbf{h}, \boldsymbol{\phi}, \boldsymbol{\gamma}', \boldsymbol{\gamma}''] = \prod_{s \in \mathbb{N}_T} \boldsymbol{\omega}_1 \mathbf{P}_{s,1} \left[\prod_{i=2}^{T} \mathbf{G}_{i-1} \mathbf{P}_{s,i} \right] \mathbf{1}_4$$

$$\boldsymbol{\omega}_1 = \begin{bmatrix} 1-\psi_1 & 0 & \psi_1(1-h_1) & \psi_1 h_1 \end{bmatrix},$$

$$\mathbf{P}_{s,t} = \begin{bmatrix} 1-I(x_{s,t}>0) & 0 & 0 & 0 \\ 0 & 1-I(x_{s,t}>0) & 0 & 0 \\ 0 & 0 & 1-I(x_{s,t}>0) & 0 \\ 0 & 0 & 0 & p_{s,t} \end{bmatrix},$$

$$\mathbf{G}_t = \begin{bmatrix} 1-\psi_t & 0 & \psi_t(1-h_t) & \psi_t h_t \\ 0 & 1 & 0 & 0 \\ 0 & 1-\phi_t & \phi_t \gamma'_t & \phi_t(1-\gamma'_t) \\ 0 & 1-\phi_t & \phi_t \gamma''_t & \phi_t(1-\gamma''_t) \end{bmatrix},$$

$$\mathbf{1}_4 = \begin{bmatrix} 1 & 1 & 1 & 1 \end{bmatrix}^{\mathrm{T}},$$

$$p_{s,t} = [x_{s,t} \mid \lambda_{s,t}],$$

$$[\mathbf{Y}, \mathbf{u}, \mathbf{e} \mid \mathbf{X}] = \prod_{t=1}^{T} I\left(\sum_{s \in \mathbb{M}_t} x_{s,t} - y_{s,t} = e_t \right) \times I\left(\sum_{s \notin \mathbb{M}_t} x_{s,t} = u_t \right),$$

$\mathbb{N}_t$ is the set of all individuals that were a member of the population at any time prior to survey t, ψ_t is the probability that an individual enters the population between sighting occasion $t-1$ and t (and was alive and not a permanent emigrant during sighting occasion t), and h_t is the conditional probability that an individual that entered the population between sighting occasion $t-1$ and t was within the study area (given alive and not a permanent emigrant).

Equation 5 can only be evaluated by solving a multidimensional summation over the true (but unobserved) sighting frequencies ($\mathbf{X}$). The situation is complicated further because both $\mathbf{X}$ and $\mathbf{Z}$ are matrices of unknown dimension $|\mathbb{N}_T| \times T$. This makes maximum likelihood estimation difficult; one could instead adopt a Bayesian perspective and use Markov chain Monte Carlo to evaluate a data-augmented posterior distribution (e.g. McClintock et al. 2014; Rankin et al. 2016).

To accomplish this, we first augment the data with $N^* >> |\mathbb{N}_T|$ sighting histories constituting a "superpopulation" of individuals, such that the augmented $\mathbf{X} = (x_{s,t})$ and $\mathbf{Z} = (z_{s,t})$ matrix sizes are $N^* \times T$. We define $q_{s,t} \in \{1, 2, 3, 4\}$ as a latent categorical variable indicating the state of individual s at time t, where $q_{s,t} = 1$ corresponds to individuals that have not yet entered the population, $q_{s,t} = 2$ corresponds to individuals that have died or permanently emigrated from the population, $q_{s,t} = 3$ corresponds to individuals that were alive and not permanent emigrants but were temporally unobservable (e.g., off the study area), and $q_{s,t} = 4$ corresponds to individuals that were alive, not permanent emigrants, and observable (e.g., on the study area). Assuming state transitions are first-order Markov, we have

$$\Pr(q_{s,t} = j \mid q_{s,t-1} = i) = G_t[i, j]$$

Under this formulation, the observable population size can be derived as $N_t =$

$\sum_{i=1}^{N^*} I(q_{s,t} = 4)$ and the total population size as $N_t + \sum_{i=1}^{N^*} I(q_{s,t} = 3)$.

To account for the fact that marked individuals must have already entered the population (i.e., $q_{s,t} > 1$ for $s \in \mathbb{M}_t$), we define $m_{s,t} \in \{0, 1\}$ as an indicator for the mark status (1=marked, 0=unmarked) of individual s at time t. We assume $m_{s,t} \mid \mathbf{q}_s, c_t \sim \text{Bernoulli}(\pi_{s,t})$, where

$$\pi_{s,t} = \begin{cases} 1 & \text{if } m_{s,t-1} = 1 \\ c_t & \text{if } m_{s,t-1} = 0 \text{ and } q_{s,t-1} > 2 \\ c_t & \text{if } q_{s,t-1} = 1 \text{ and } q_{s,t} > 1 \\ 0 & \text{otherwise} \end{cases},$$

$m_{s,0} = 0$, $q_{s,0} = 1$, and c_t is the probability that an unmarked individual becomes marked between times $t - 1$ and t. Note that for simplicity we assume no tag loss.

The resulting joint posterior distribution is:

$$\begin{aligned}
&[\mathbf{X}, \mathbf{Q}, \mathbf{Z}, \boldsymbol{\alpha}, \boldsymbol{\sigma}, \boldsymbol{\psi}, \mathbf{h}, \boldsymbol{\phi}, \boldsymbol{\gamma}', \boldsymbol{\gamma}'', \boldsymbol{c} \mid \mathbf{Y}, \mathbf{e}, \mathbf{u}, \mathbf{M}] \propto \\
&\quad [\mathbf{X} \mid \mathbf{Q}, \mathbf{Z}, \boldsymbol{\alpha}, \boldsymbol{\sigma}] \, [\mathbf{Y}, \mathbf{u}, \mathbf{e} \mid \mathbf{X}] \\
&\quad \times [\mathbf{M} \mid \mathbf{Q}, \mathbf{c}] \, [\mathbf{Q} \mid \boldsymbol{\psi}, \mathbf{h}, \boldsymbol{\phi}, \boldsymbol{\gamma}', \boldsymbol{\gamma}''] \\
&\quad \times [\mathbf{Z}] \, [\boldsymbol{\alpha}] \, [\boldsymbol{\sigma}] \, [\boldsymbol{\psi}] \, [\mathbf{h}] \, [\boldsymbol{\phi}] \, [\boldsymbol{\gamma}'] \, [\boldsymbol{\gamma}''] \, [\boldsymbol{c}] \,,
\end{aligned}$$

where

$$[\mathbf{X} \mid \mathbf{Q}, \mathbf{Z}, \boldsymbol{\alpha}, \boldsymbol{\sigma}] = \prod_{s=1}^{N^*} \prod_{t=1}^{T} [x_{s,t} \mid q_{s,t}, z_{s,t}, \alpha_t, \sigma_t] \,,$$

$$x_{s,t} \mid q_{s,t}, z_{s,t}, \alpha_t, \sigma_t \sim \text{Poisson}(\lambda_{s,t} I(q_{s,t} = 4)),$$

and $\lambda_{s,t} = \exp(\alpha_t + \sigma_t z_{s,t})$. Note that for the "across" heterogeneity model, we have $z_{s,1} = z_{s,2} = \cdots = z_{s,T}$.

A version of this Bayesian model can be relatively easily coded in JAGS and implemented using R package `rjags` (Plummer 2016). For completeness, we provide

example code in the Supporting Information for motivated readers. However, in our limited explorations of this data-augmented complete data likelihood under various prior distributions, we have not found the MCMC algorithm to efficiently explore the parameter space (due to what appears to be very poor mixing) and have therefore found application and further investigation of its properties impractical (particularly for large T or N^*). Such model-fitting challenges were our primary motivation for developing a composite likelihood that could be easily implemented by practitioners using Program MARK.

Web Appendix B: Definitions

Table B1. Definitions of parameters, latent variables, data, and other quantities for the ziPNE composite likelihood.

Parameters	Definition
U_t	Number of unmarked individuals in the observable population at time t
α_t	log-scale mean for sighting rate ($\lambda_{s,t}$)
σ_t	log-scale standard deviation for sighting rate ($\lambda_{s,t}$)
r_t	conditional probability that a mark is individually identified
w_t	probability that a newly marked individual was alive and not a permanent emigrant at time t
g_t	conditional probability that a newly marked individual was within the study area at time t
ϕ_t	apparent survival probability from time t to $t+1$
γ'_t	transition probability from an observable state to an unobservable state
γ''_t	transition probability from an unobservable state to an unobservable state

Latent variables	Definition
$z_{s,t}$	individual-level effects for sighting rate ($\lambda_{s,t}$)

Data	Definition
$y_{s,t}$	Number of identified sightings for marked individual s at time t
e_t	Total number of unidentified mark sightings at time t
u_t	Total number of unmarked individual sightings at time t
R_t	Number of (unmarked) individuals that were newly marked between times $t-1$ and t
M_t	Number of marked individuals ever released into the population prior to survey t
$\mathbb{M}_t$	Set of M_t marked individuals ever released into the population prior to survey t

Other quantities	Definition
$\lambda_{s,t} = \exp(\alpha_t + \sigma_t z_{s,t})$	Poisson log-normal sighting rate for individual s at time t
λ_t	mean sighting rate at time t
$p_{s,t}$	Shorthand for the probability mass function of $y_{s,t}$
p_t	Probability of being detected at least once at time t
N_t	Number of individuals in the observable population

Web Appendix C: Additional simulation results

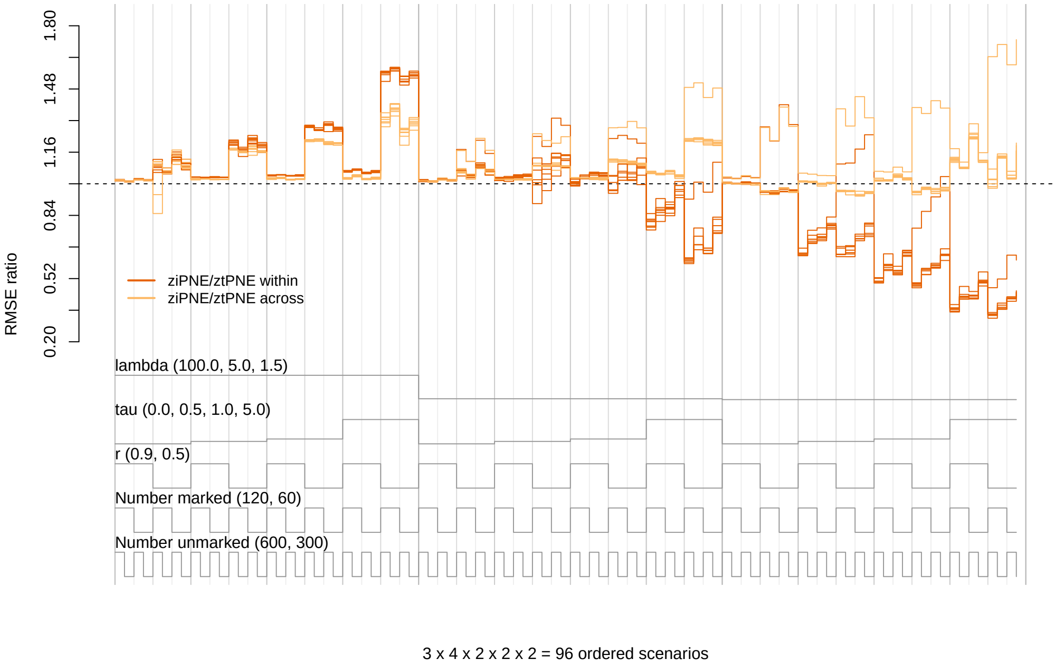

Figure C1. Nested loop plots of ratio of root mean squared errors (RMSE ratio) of the zero-inflated (ziPNE) and zero-truncated (ztPNE) Poisson log-normal abundance $(N_t, t = 1, \ldots, 7)$ estimators based on 192 simulated scenarios. Scenarios are ordered from outer to inner loops by $\lambda_t \in \{100, 5, 1.5\}$ ("lambda"), $\tau \in \{0, 0.5, 1, 5\}$ ("tau"), $r \in \{0.9, 0.5\}$, $\mathrm{E}(R_1) \in \{120, 60\}$ ("Number marked"), and $\mathrm{E}(U_1) \in \{600, 300\}$ ("Number unmarked") such that the left- and right-most scenarios are the best- and worst-case sampling scenarios, respectively. RMSE ratios were calculated as RMSE(ziPNE)/RMSE(ztPNE) for the within heterogeneity models (*dark orange*) and the across heterogeneity models (*light orange*).

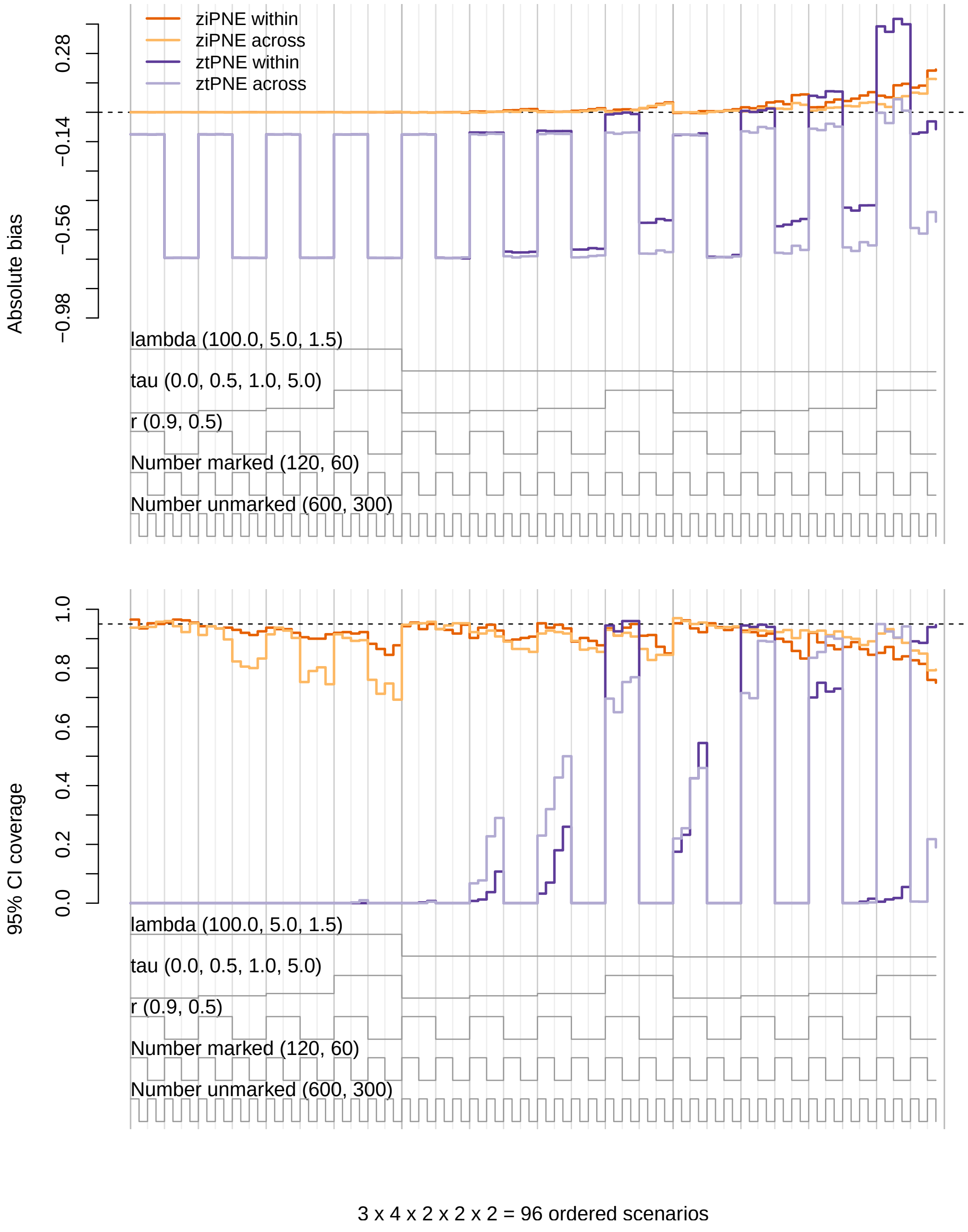

Figure C2. Nested loop plots of absolute bias (top panel) and 95% confidence interval coverage (bottom panel) of α estimators based on 192 simulated scenarios. Scenarios are ordered from outer to inner loops by $\lambda_t \in \{100, 5, 1.5\}$ ("lambda"), $\tau \in \{0, 0.5, 1, 5\}$ ("tau"), $r \in \{0.9, 0.5\}$, $\mathrm{E}(R_1) \in \{120, 60\}$ ("Number marked"), and $\mathrm{E}(U_1) \in \{600, 300\}$ ("Number unmarked") such that the left- and right-most scenarios are the best- and worst-case sampling scenarios, respectively. Estimators include the zero-inflated Poisson log-normal estmator (*dark orange* = ziPNE within, *light orange* = ziPNE across) and the zero-truncated Poisson log-normal estimator (*dark purple* = ztPNE within, *light purple* = ztPNE across).

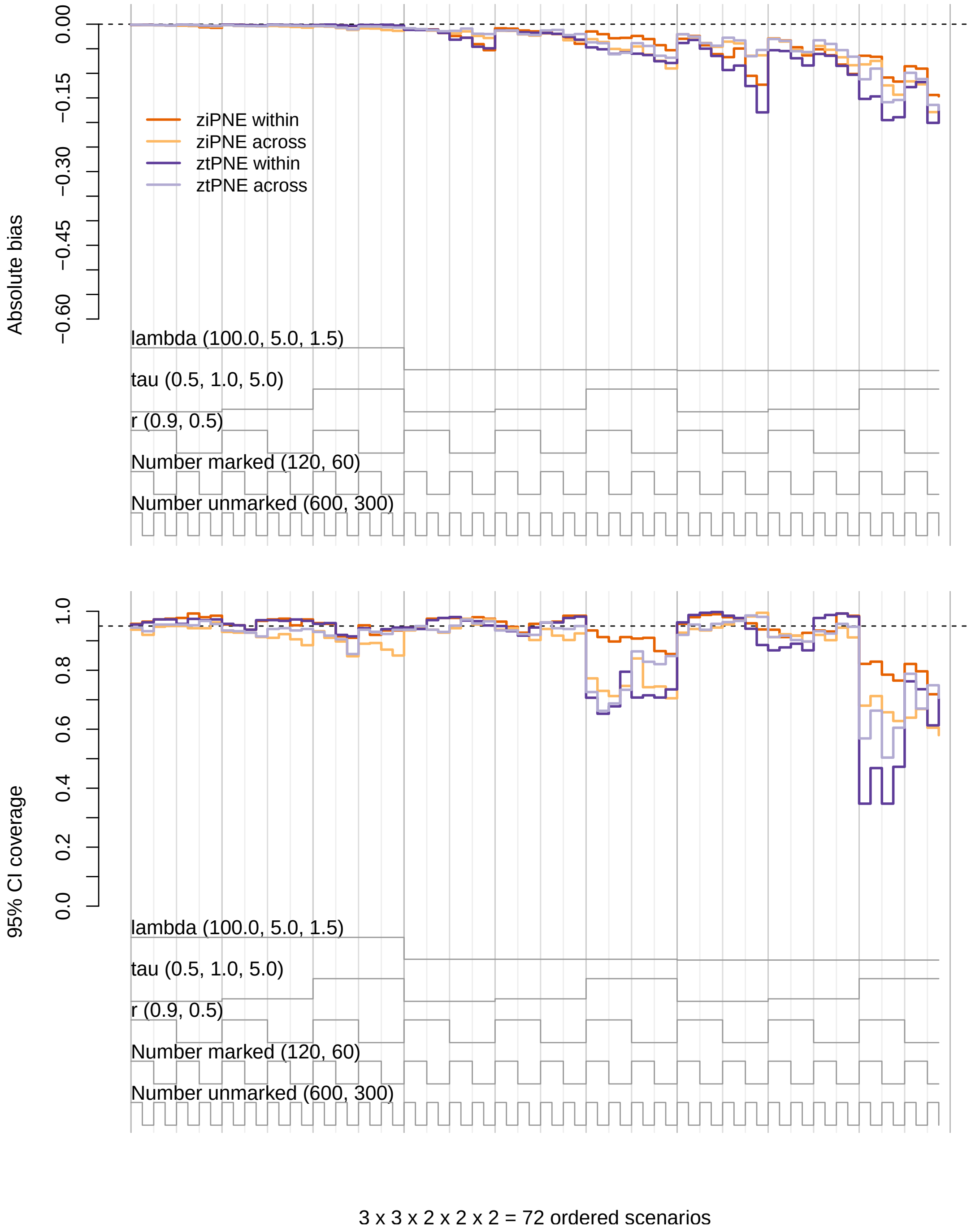

Figure C3. Nested loop plots of absolute bias (top panel) and 95% confidence interval coverage (bottom panel) of σ estimators based on 144 simulated scenarios. Scenarios are ordered from outer to inner loops by $\lambda_t \in \{100, 5, 1.5\}$ ("lambda"), $\tau \in \{0.5, 1, 5\}$ ("tau"), $r \in \{0.9, 0.5\}$, $\mathrm{E}(R_1) \in \{120, 60\}$ ("Number marked"), and $\mathrm{E}(U_1) \in \{600, 300\}$ ("Number unmarked") such that the left- and right-most scenarios are the best- and worst-case sampling scenarios, respectively. Estimators include the zero-inflated Poisson log-normal estmator (*dark orange* = ziPNE within, *light orange* = ziPNE across) and the zero-truncated Poisson log-normal estimator (*dark purple* = ztPNE within, *light purple* = ztPNE across).

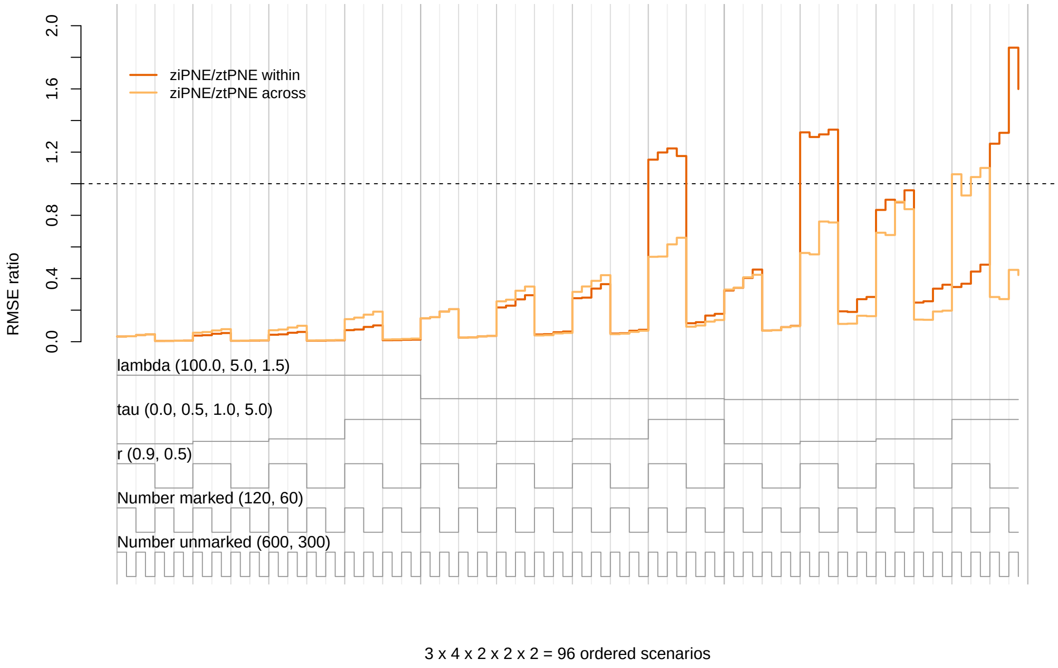

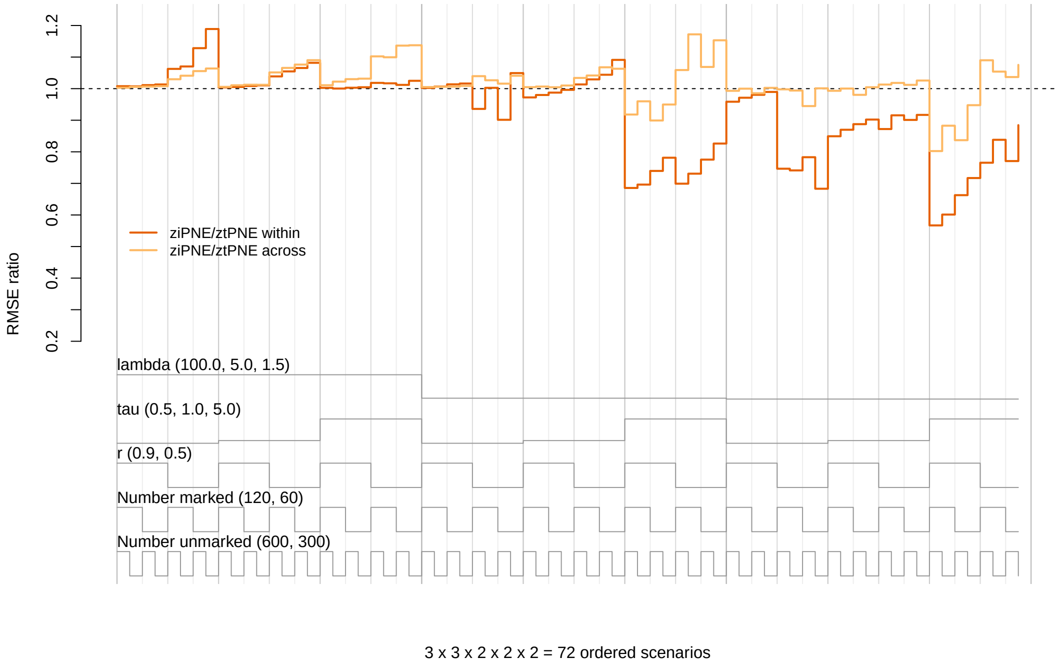

Figure C4. Nested loop plots of ratio of root mean squared errors (RMSE ratio) of the zero-inflated (ziPNE) and zero-truncated (ztPNE) Poisson log-normal α (top panel) and σ (lower panel) estimators based on simulated scenarios. Scenarios are ordered from outer to inner loops by $\lambda_t \in \{100, 5, 1.5\}$ ("lambda"), $\tau \in \{0, 0.5, 1, 5\}$ ("tau"), $r \in \{0.9, 0.5\}$, $\mathrm{E}(R_1) \in \{120, 60\}$ ("Number marked"), and $\mathrm{E}(U_1) \in \{600, 300\}$ ("Number unmarked") such that the left- and right-most scenarios are the best- and worst-case sampling scenarios, respectively. RMSE ratios were calculated as RMSE(ziPNE)/RMSE(ztPNE) for the within heterogeneity models (*dark orange*) and the across heterogeneity models (*light orange*).

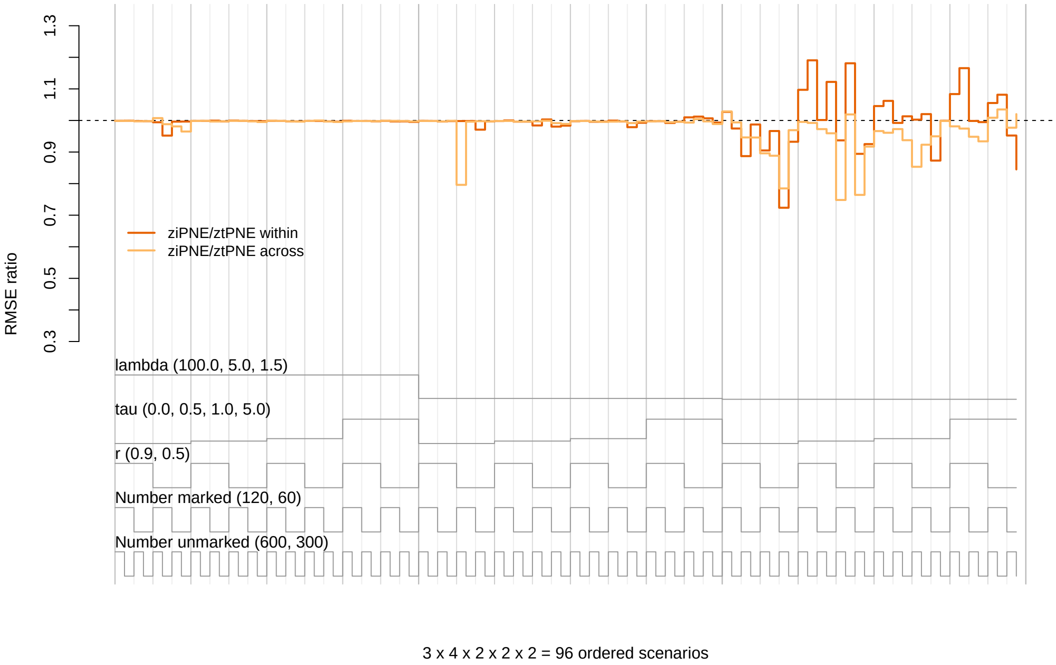

Figure C5. Nested loop plots of ratio of root mean squared errors (RMSE ratio) of the zero-inflated (ziPNE) and zero-truncated (ztPNE) Poisson log-normal apparent survival (ϕ) estimators based on 192 simulated scenarios. Scenarios are ordered from outer to inner loops by $\lambda_t \in \{100, 5, 1.5\}$ ("lambda"), $\tau \in \{0, 0.5, 1, 5\}$ ("tau"), $r \in \{0.9, 0.5\}$, $\mathrm{E}(R_1) \in \{120, 60\}$ ("Number marked"), and $\mathrm{E}(U_1) \in \{600, 300\}$ ("Number unmarked") such that the left- and right-most scenarios are the best- and worst-case sampling scenarios, respectively. RMSE ratios were calculated as RMSE(ziPNE)/RMSE(ztPNE) for the within heterogeneity models (*dark orange*) and the across heterogeneity models (*light orange*).

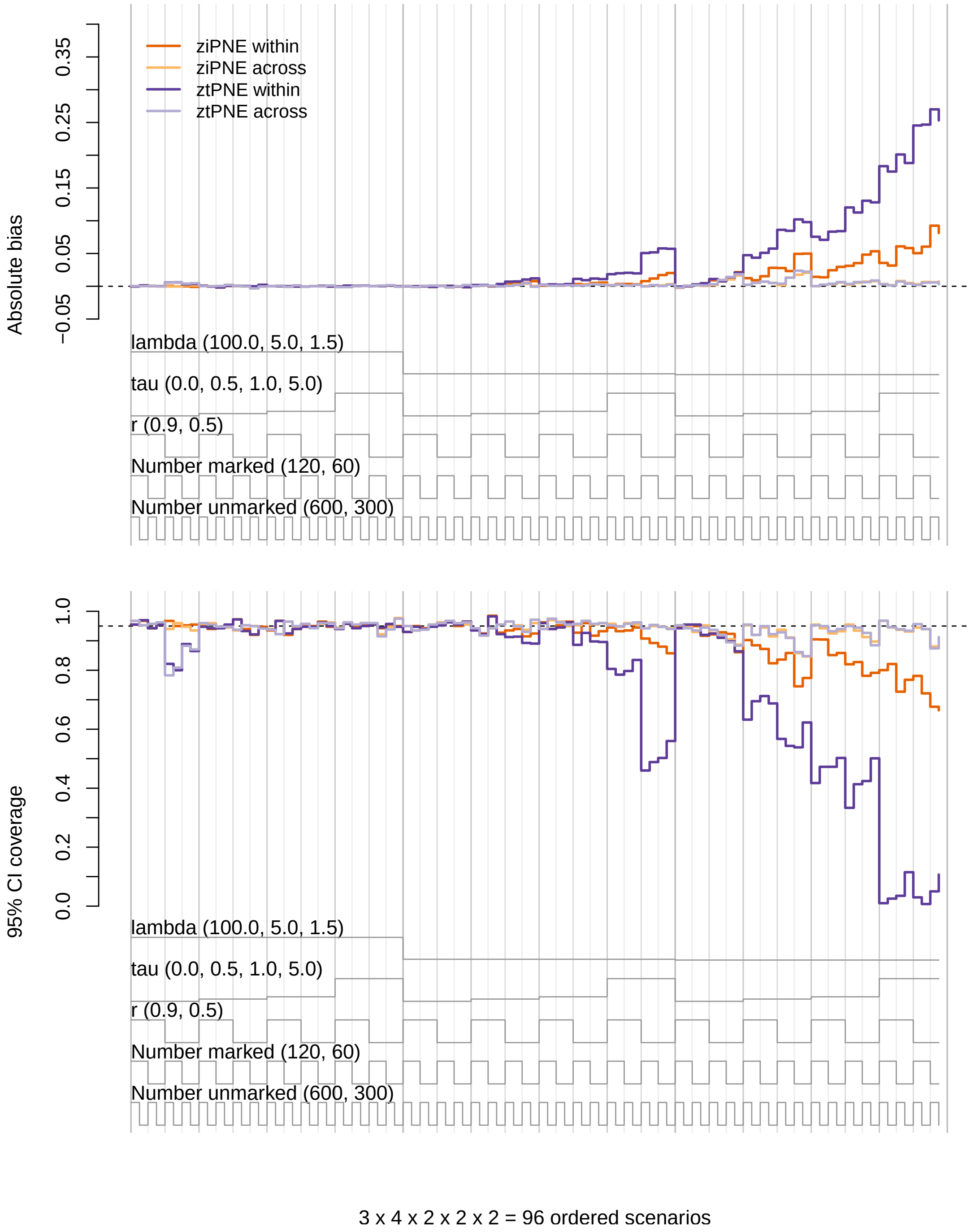

Figure C6. Nested loop plots of absolute bias (top panel) and 95% confidence interval coverage (bottom panel) of γ'' estimators based on 192 simulated scenarios. Scenarios are ordered from outer to inner loops by $\lambda_t \in \{100, 5, 1.5\}$ ("lambda"), $\tau \in \{0, 0.5, 1, 5\}$ ("tau"), $r \in \{0.9, 0.5\}$, $\mathrm{E}(R_1) \in \{120, 60\}$ ("Number marked"), and $\mathrm{E}(U_1) \in \{600, 300\}$ ("Number unmarked") such that the left- and right-most scenarios are the best- and worst-case sampling scenarios, respectively. Estimators include the zero-inflated Poisson log-normal estmator (*dark orange* = ziPNE within, *light orange* = ziPNE across) and the zero-truncated Poisson log-normal estimator (*dark purple* = ztPNE within, *light purple* = ztPNE across).

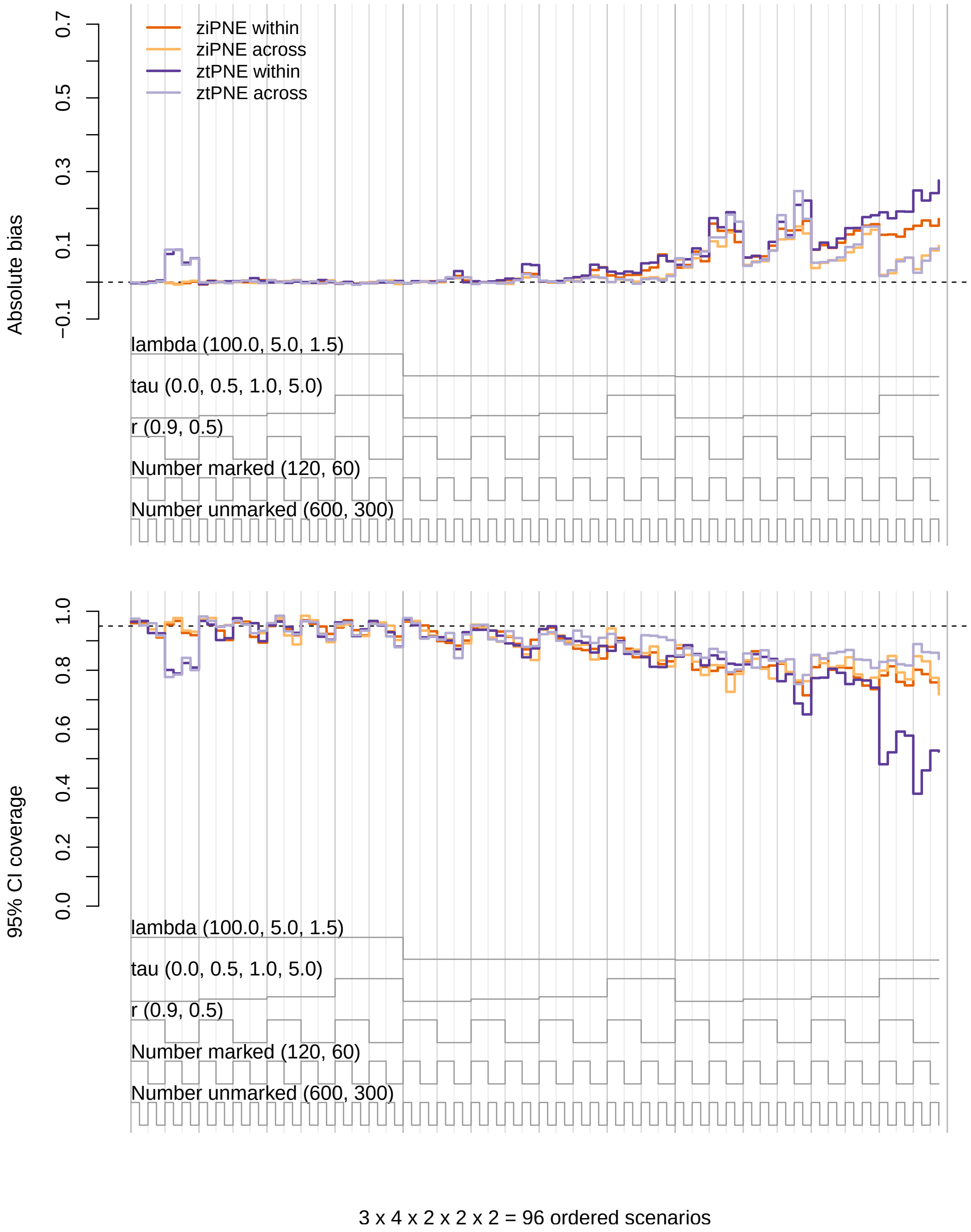

Figure C7. Nested loop plots of absolute bias (top panel) and 95% confidence interval coverage (bottom panel) of γ' estimators based on 192 simulated scenarios. Scenarios are ordered from outer to inner loops by $\lambda_t \in \{100, 5, 1.5\}$ ("lambda"), $\tau \in \{0, 0.5, 1, 5\}$ ("tau"), $r \in \{0.9, 0.5\}$, $\mathrm{E}(R_1) \in \{120, 60\}$ ("Number marked"), and $\mathrm{E}(U_1) \in \{600, 300\}$ ("Number unmarked") such that the left- and right-most scenarios are the best- and worst-case sampling scenarios, respectively. Estimators include the zero-inflated Poisson log-normal estmator (*dark orange* = ziPNE within, *light orange* = ziPNE across) and the zero-truncated Poisson log-normal estimator (*dark purple* = ztPNE within, *light purple* = ztPNE across).

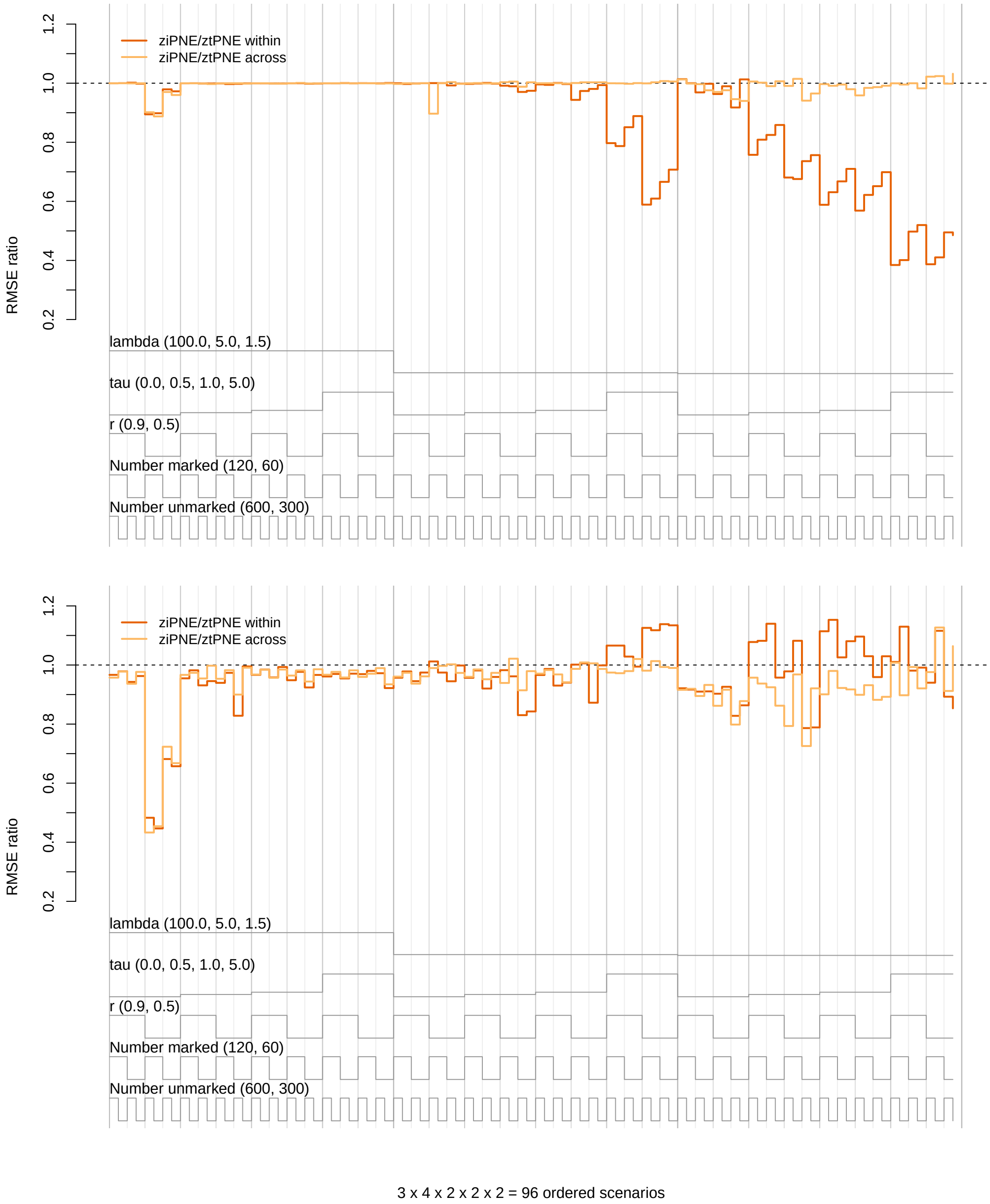

Figure C8. Nested loop plots of ratio of root mean squared errors (RMSE ratio) of the zero-inflated (ziPNE) and zero-truncated (ztPNE) Poisson log-normal γ'' (top panel) and γ' (lower panel) estimators based on 192 simulated scenarios. Scenarios are ordered from outer to inner loops by $\lambda_t \in \{100, 5, 1.5\}$ ("lambda"), $\tau \in \{0, 0.5, 1, 5\}$ ("tau"), $r \in \{0.9, 0.5\}$, $\mathrm{E}(R_1) \in \{120, 60\}$ ("Number marked"), and $\mathrm{E}(U_1) \in \{600, 300\}$ ("Number unmarked") such that the left- and right-most scenarios are the best- and worst-case sampling scenarios, respectively. RMSE ratios were calculated as RMSE(ziPNE)/RMSE(ztPNE) for the within heterogeneity models (*dark orange*) and the across heterogeneity models (*light orange*).

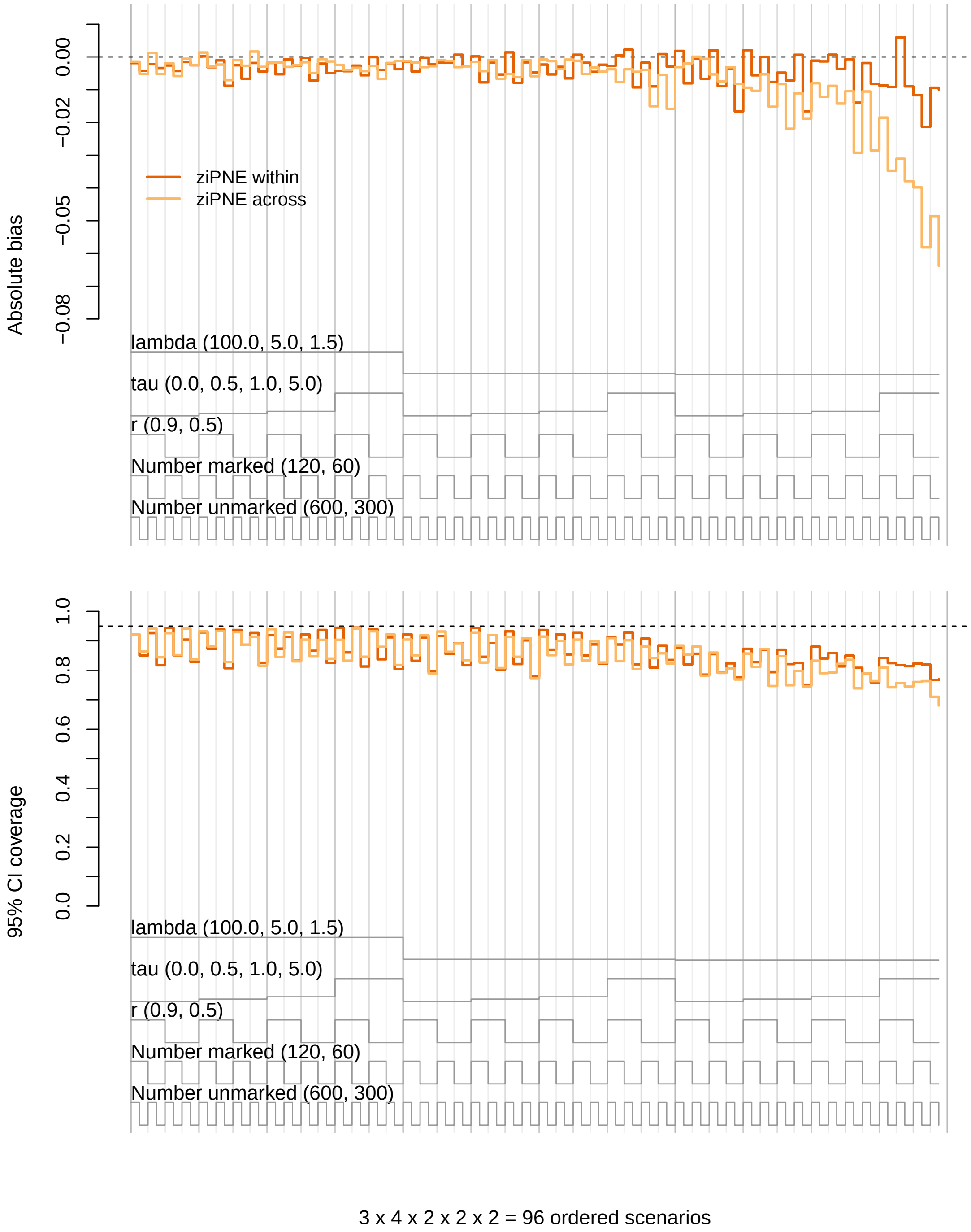

Figure C9. Nested loop plots of absolute bias (top panel) and 95% confidence interval coverage (bottom panel) of w estimators based on 192 simulated scenarios. Scenarios are ordered from outer to inner loops by $\lambda_t \in \{100, 5, 1.5\}$ ("lambda"), $\tau \in \{0, 0.5, 1, 5\}$ ("tau"), $r \in \{0.9, 0.5\}$, $\mathrm{E}(R_1) \in \{120, 60\}$ ("Number marked"), and $\mathrm{E}(U_1) \in \{600, 300\}$ ("Number unmarked") such that the left- and right-most scenarios are the best- and worst-case sampling scenarios, respectively. Estimators include the zero-inflated Poisson log-normal estmator with "within" heterogeneity (*dark orange*) and "across" heterogeneity (*light orange*).

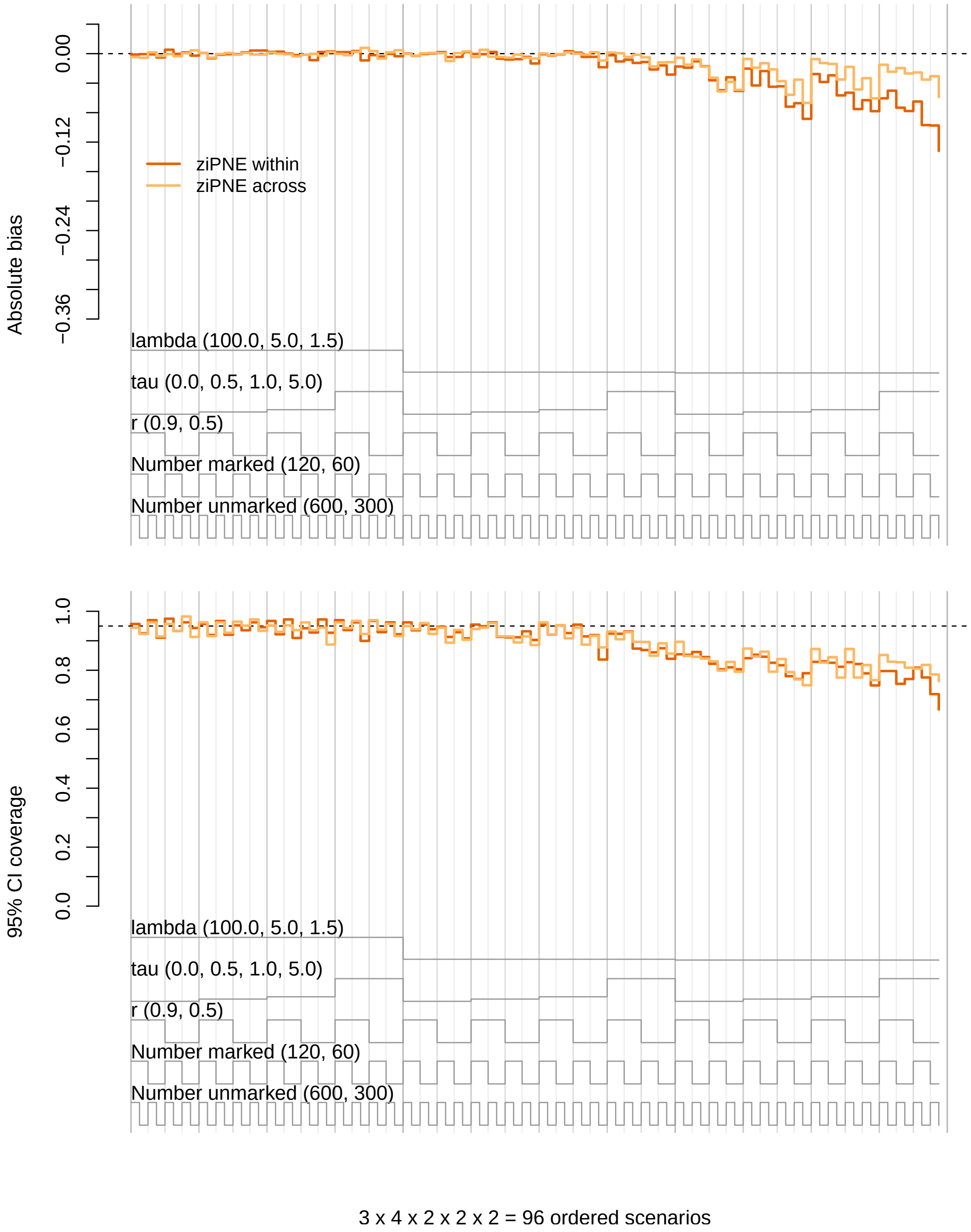

Figure C10. Nested loop plots of absolute bias (top panel) and 95% confidence interval coverage (bottom panel) of g estimators based on 192 simulated scenarios. Scenarios are ordered from outer to inner loops by $\lambda_t \in \{100, 5, 1.5\}$ ("lambda"), $\tau \in \{0, 0.5, 1, 5\}$ ("tau"), $r \in \{0.9, 0.5\}$, $\mathrm{E}(R_1) \in \{120, 60\}$ ("Number marked"), and $\mathrm{E}(U_1) \in \{600, 300\}$ ("Number unmarked") such that the left- and right-most scenarios are the best- and worst-case sampling scenarios, respectively. Estimators include the zero-inflated Poisson log-normal estmator with "within" heterogeneity (*dark orange*) and "across" heterogeneity (*light orange*).

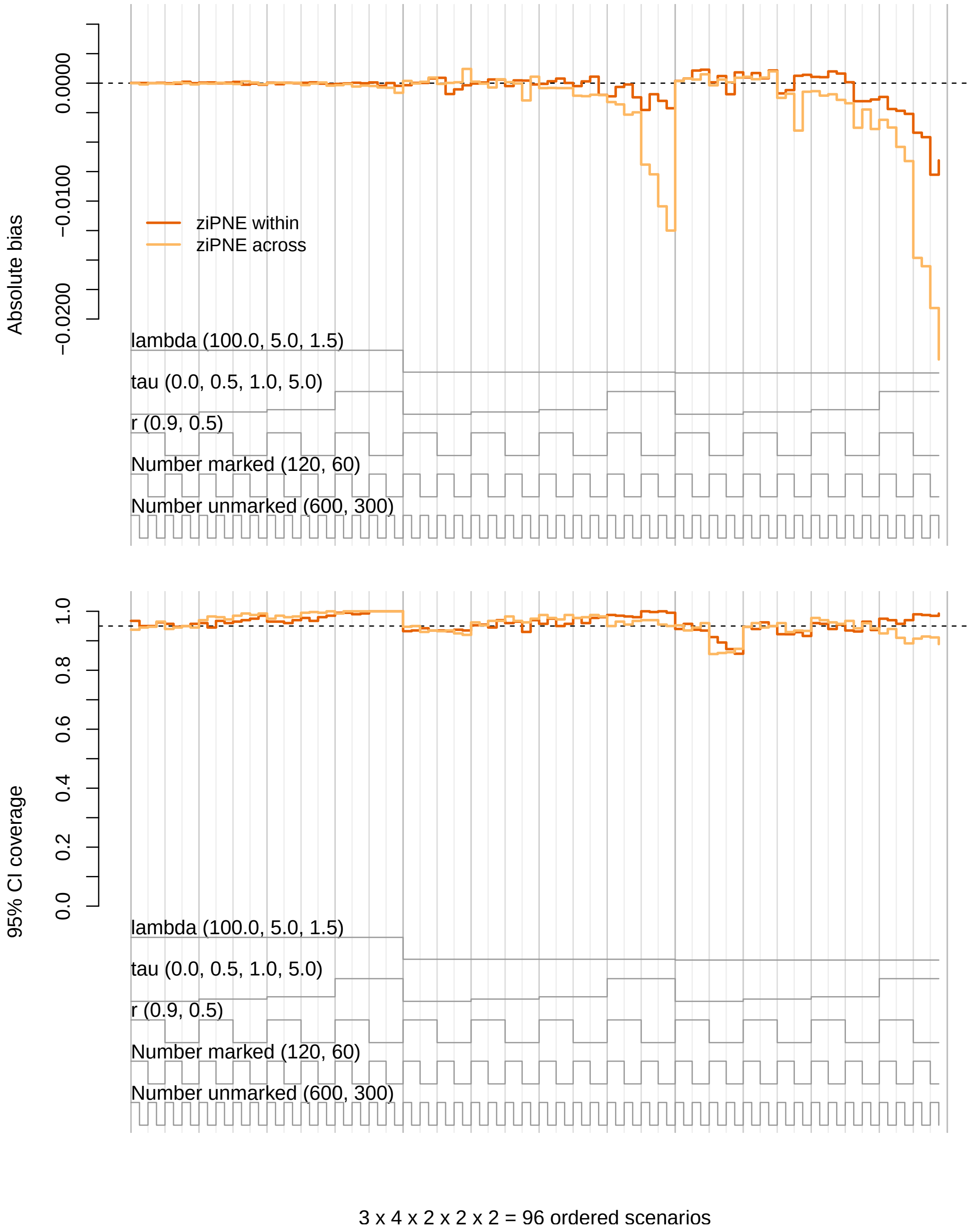

Figure C11. Nested loop plots of percent relative bias (top panel) and 95% confidence interval coverage (bottom panel) of r estimators based on 192 simulated scenarios. Scenarios are ordered from outer to inner loops by $\lambda_t \in \{100, 5, 1.5\}$ ("lambda"), $\tau \in \{0, 0.5, 1, 5\}$ ("tau"), $r \in \{0.9, 0.5\}$, $\mathrm{E}(R_1) \in \{120, 60\}$ ("Number marked"), and $\mathrm{E}(U_1) \in \{600, 300\}$ ("Number unmarked") such that the left- and right-most scenarios are the best- and worst-case sampling scenarios, respectively. Estimators include the zero-inflated Poisson log-normal estmator with "within" heterogeneity (*dark orange*) and "across" heterogeneity (*light orange*).

Web Appendix D: Additional New Zealand robin example results

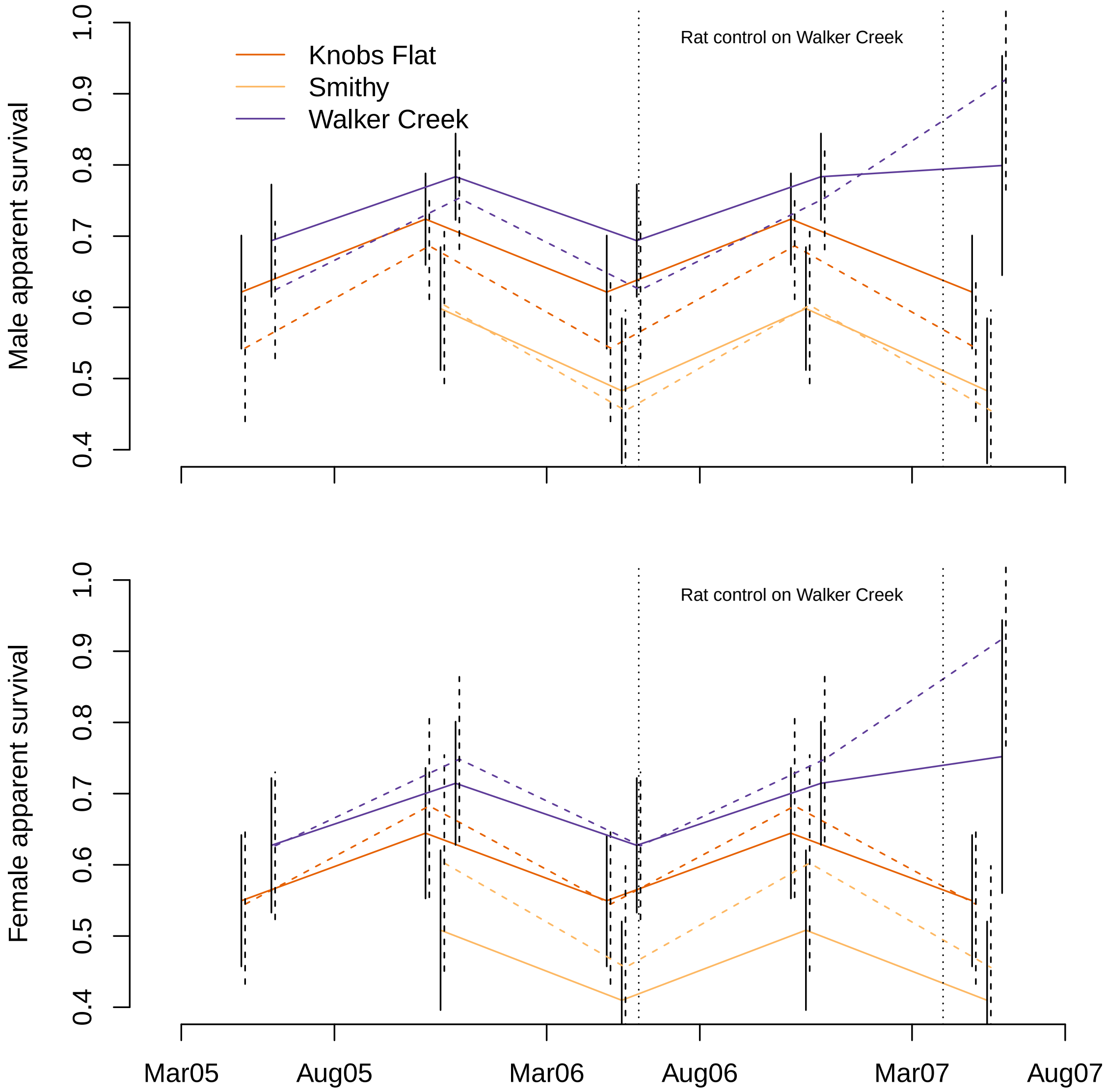

Figure D1. Model-averaged four-month apparent survival (ϕ) estimates ($\pm$SE) between March 2005 and August 2007 for three New Zealand Robin populations (*dark orange* = Knobs Flat, *light orange* = Smithy, and *dark purple* = Walker Creek) in Fiordland National Park, New Zealand. Estimates are for males (top panel) and females (bottom panel) that were first captured as juveniles based on the zero-inflated model (ziPNE; solid lines) and the zero-truncated model (ztPNE; dashed lines). The longer intervals between August and March encompass the breeding season. Vertical dotted lines indicate a period of rat control on the Walker Creek study area.

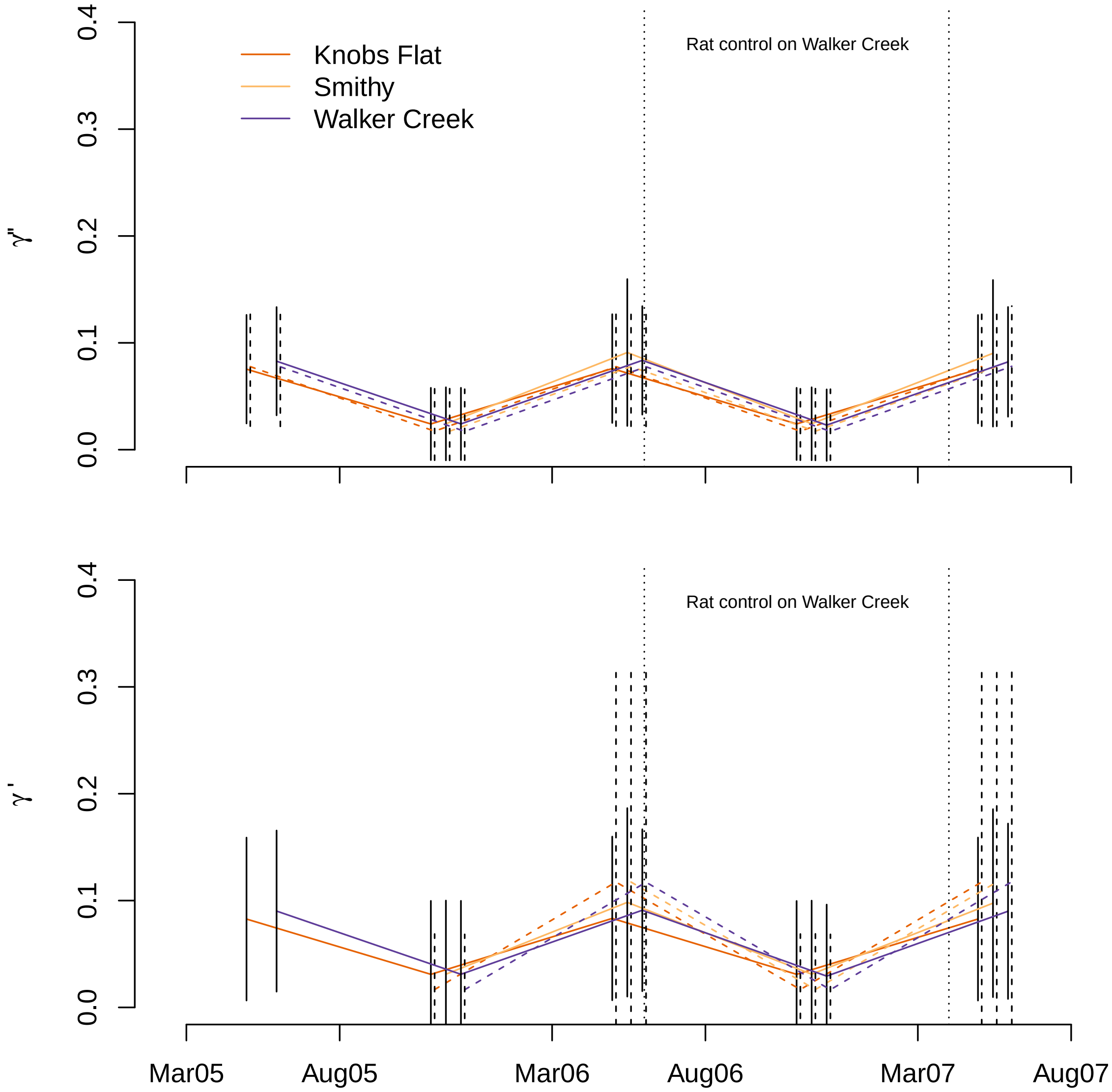

Figure D2. Model-averaged four-month estimates (±SE) for transition probabilities from an observable to unobservable state (γ''; top panel) and from an unobservable to unobservable state (γ'; bottom panel) between March 2005 and August 2007 for three New Zealand Robin populations (*dark orange* = Knobs Flat, *light orange* = Smithy, and *dark purple* = Walker Creek) in Fiordland National Park, New Zealand. Estimates are for individuals first captured as adults based on the zero-inflated model (ziPNE; solid lines) and the zero-truncated model (ztPNE; dashed lines). The longer intervals between August and March encompass the breeding season. Vertical dotted lines indicate a period of rat control on the Walker Creek study area.

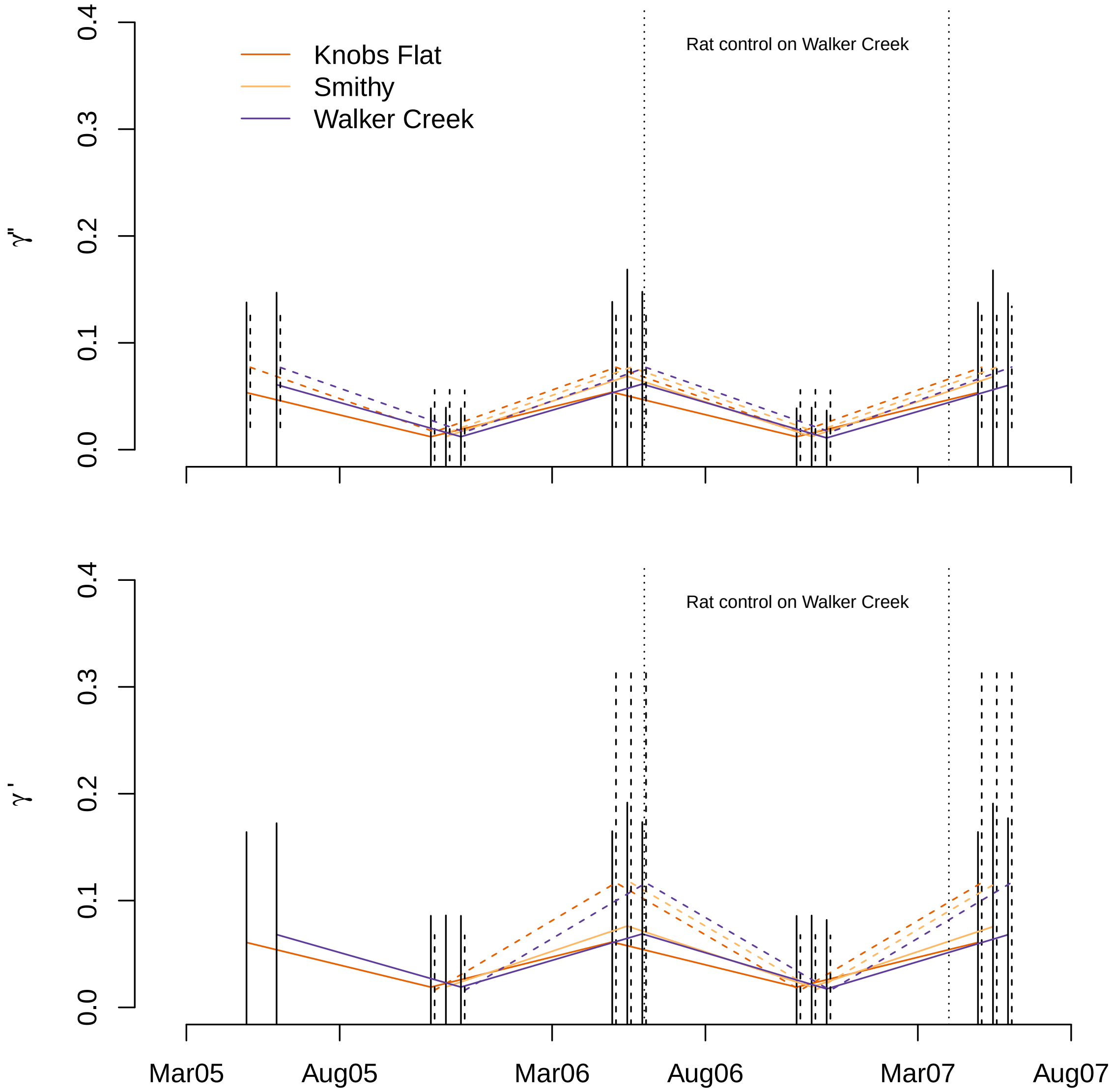

Figure D3. Model-averaged four-month estimates (±SE) for transition probabilities from an observable to unobservable state (γ''; top panel) and from an unobservable to unobservable state (γ'; bottom panel) between March 2005 and August 2007 for three New Zealand Robin populations (*dark orange* = Knobs Flat, *light orange* = Smithy, and *dark purple* = Walker Creek) in Fiordland National Park, New Zealand. Estimates are for adults first captured as juveniles based on the zero-inflated model (ziPNE; solid lines) and the zero-truncated model (ztPNE; dashed lines). The longer intervals between August and March encompass the breeding season. Vertical dotted lines indicate a period of rat control on the Walker Creek study area.

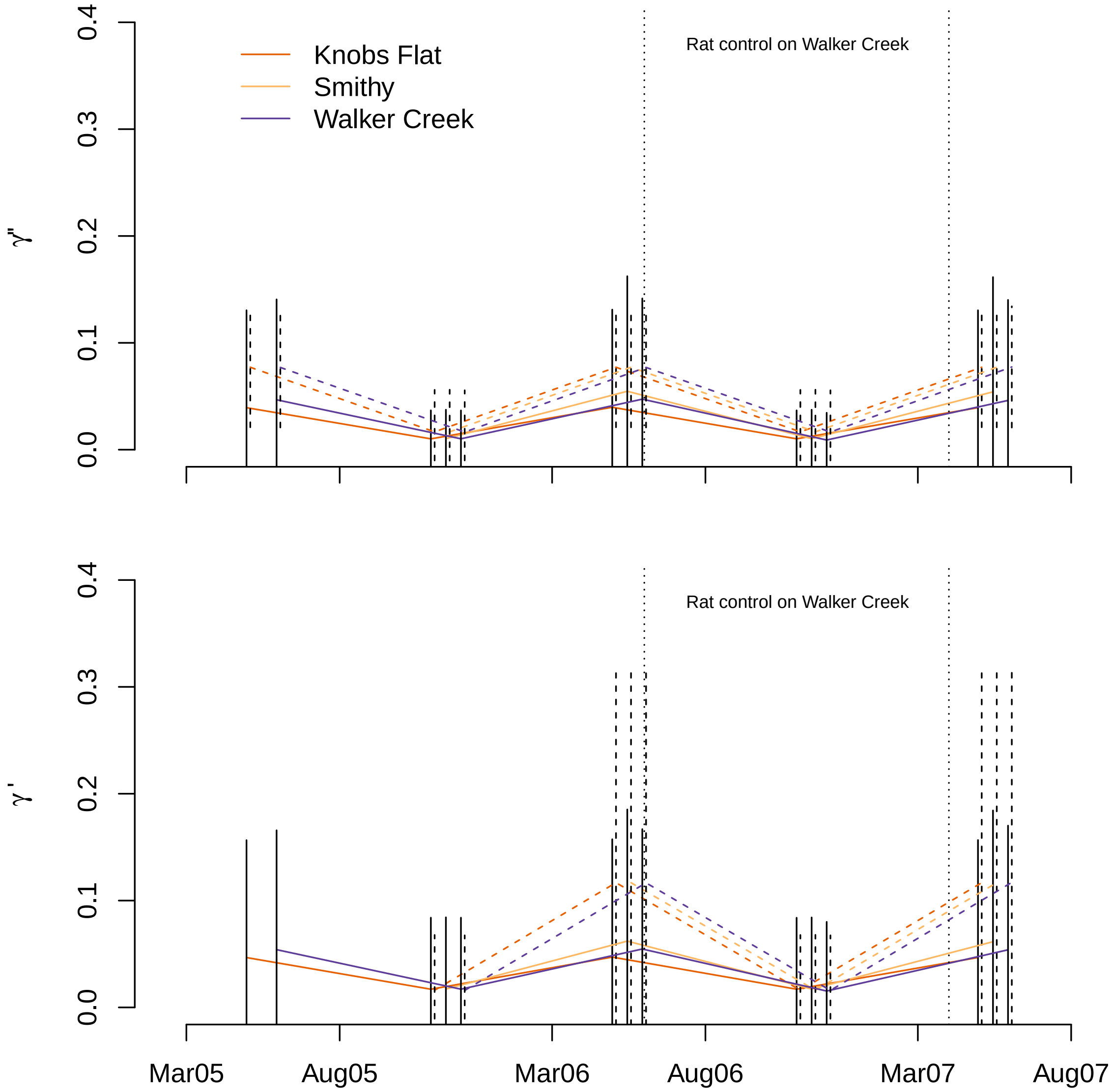

Figure D4. Model-averaged four-month estimates (±SE) for transition probabilities from an observable to unobservable state (γ''; top panel) and from an unobservable to unobservable state (γ'; bottom panel) between March 2005 and August 2007 for three New Zealand Robin populations (*dark orange* = Knobs Flat, *light orange* = Smithy, and *dark purple* = Walker Creek) in Fiordland National Park, New Zealand. Estimates are for juveniles based on the zero-inflated model (ziPNE; solid lines) and the zero-truncated model (ztPNE; dashed lines). The longer intervals between August and March encompass the breeding season. Vertical dotted lines indicate a period of rat control on the Walker Creek study area.

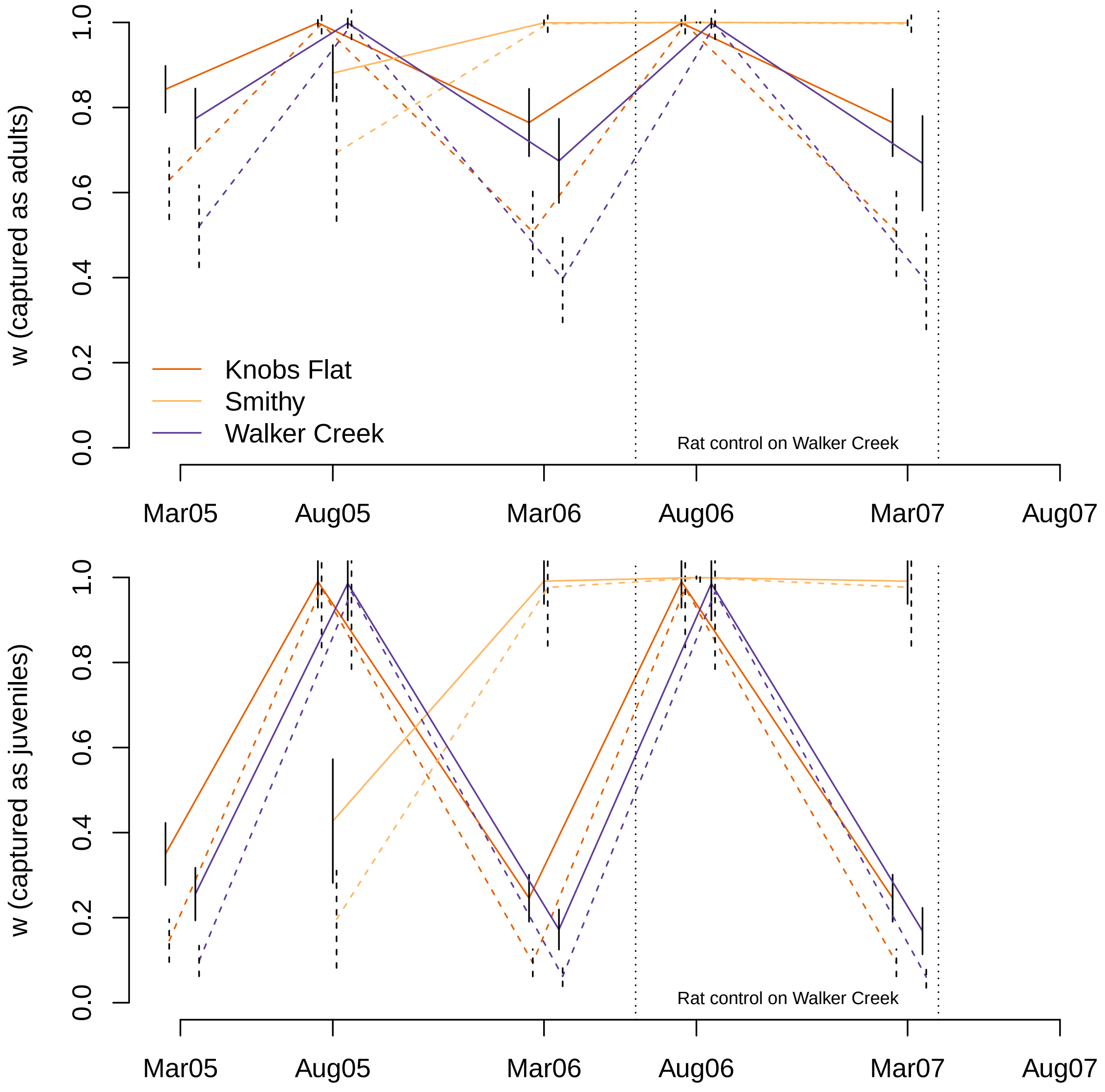

Figure D5. Model-averaged w estimates (±SE) between March 2005 and August 2007 for three New Zealand Robin populations (*dark orange* = Knobs Flat, *light orange* = Smithy, and *dark purple* = Walker Creek) in Fiordland National Park, New Zealand. Estimates are for males (solid lines) and females (dashed lines) first captured as adults (top panel) and juveniles (bottom panel) based on the zero-inflated model (ziPNE). The longer intervals between August and March encompass the breeding season. Vertical dotted lines indicate a period of rat control on the Walker Creek study area.

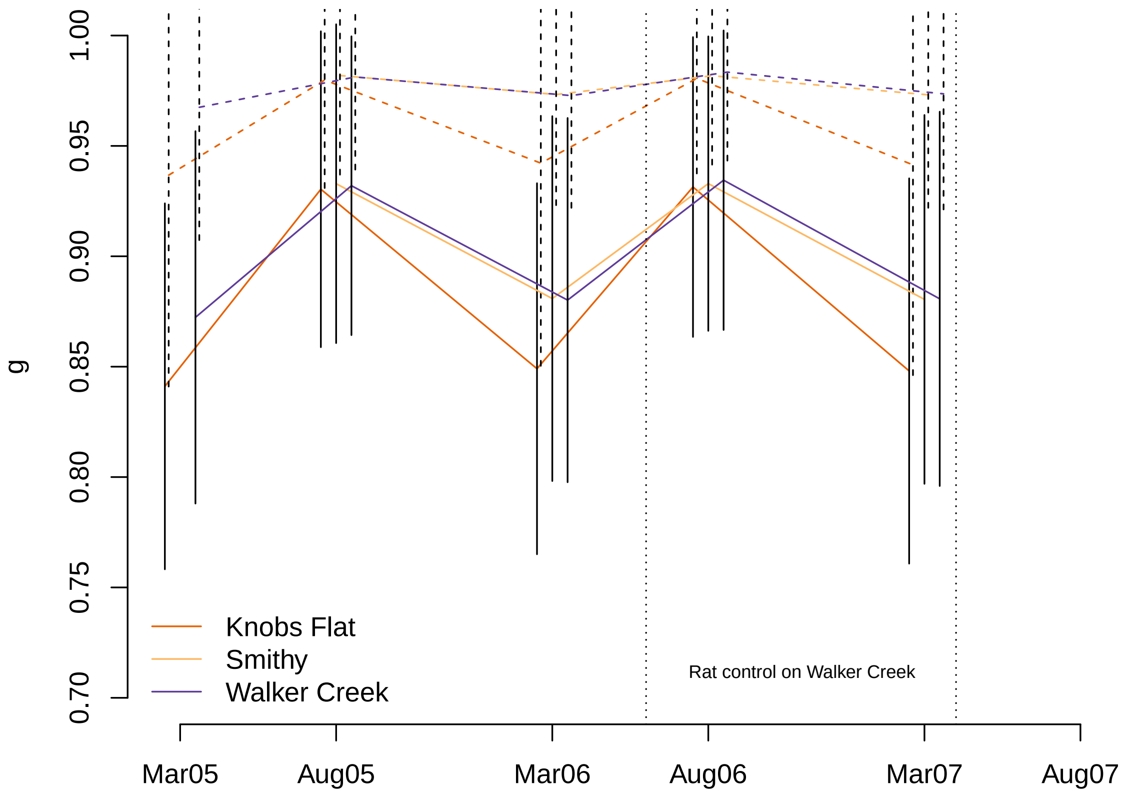

Figure D6. Model-averaged g estimates ($\pm$SE) between March 2005 and August 2007 for three New Zealand Robin populations (*dark orange* = Knobs Flat, *light orange* = Smithy, and *dark purple* = Walker Creek) in Fiordland National Park, New Zealand. Estimates are for adults (solid lines) and juveniles (dashed lines) based on the zero-inflated model (ziPNE). The longer intervals between August and March encompass the breeding season. Vertical dotted lines indicate a period of rat control on the Walker Creek study area.

model	AICc	ΔAICc	weight	K
α(A+S+R)ϕ(G+C+S+B)γ(B+C)w(G+C+S+B)g(B+C)	2442.13	0	0.06	60
α(A+S+R)ϕ(G+C+S+B)γ(C)w(G+C+S+B)g(C)	2442.15	0.02	0.06	58
α(A+S+R)ϕ(G+C+S+B+R2)γ(G+B)w(G+C+S+B+R2)g(G+B)	2443.22	1.09	0.04	64
α(S+R)ϕ(G+C+S+B)γ(C)w(G+C+S+B)g(C)	2443.22	1.09	0.04	57
α(A+S+R)ϕ(G+C+S+B+R2)γ(B+C)w(G+C+S+B+R2)g(B+C)	2443.31	1.18	0.03	62
α(A+S+R)ϕ(G+C+S+B)γ(B)w(G+C+S+B)g(B)	2443.8	1.67	0.03	58
α(A+S+R)ϕ(G+C+S+B)γ(B+A)w(G+C+S+B)g(B+A)	2443.86	1.73	0.03	60
α(A+S+R)ϕ(G+C+S+B+R2)γ(C)w(G+C+S+B+R2)g(C)	2444.06	1.93	0.02	60
α(A+S+R)ϕ(G+C+S+B+R2)γ(B+A)w(G+C+S+B+R2)g(B+A)	2444.21	2.08	0.02	62
α(A+S+R)σ(.)ϕ(G+C+S+B)γ(B+A)w(G+C+S+B)g(B+A)	2444.26	2.13	0.02	59
α(A+S+R)σ(.)ϕ(G+C+S+B)γ(.)w(G+C+S+B)g(.)	2444.33	2.2	0.02	55
α(A+S+A:S+R)ϕ(G+C+S+B)γ(C)w(G+C+S+B)g(C)	2444.38	2.25	0.02	59
α(A+S+R)ϕ(G+C+S+B+C:S:B+R2)γ(B+A)w(G+C+S+B+C:S:B+R2)g(B+A)	2444.49	2.36	0.02	64
α(A+S+A:S+R)ϕ(G+C+S+B)γ(B+C)w(G+C+S+B)g(B+C)	2444.72	2.59	0.02	61
α(A+S+R)ϕ(G+C+S+B+S:B)γ(C)w(G+C+S+B+S:B)g(C)	2444.84	2.71	0.02	60
α(A+S+R)σ(.)ϕ(G+C+S+B)γ(B)w(G+C+S+B)g(B)	2444.86	2.73	0.02	57
α(S+R)σ(.)ϕ(G+C+S+B)γ(B+A)w(G+C+S+B)g(B+A)	2444.87	2.74	0.02	58
α(A+S+R)σ(.)ϕ(G+C+S+B)γ(B+C)w(G+C+S+B)g(B+C)	2444.95	2.82	0.02	59
α(A+S+R)ϕ(G+C+S+B+C:S:B+R2)γ(B)w(G+C+S+B+C:S:B+R2)g(B)	2444.97	2.84	0.02	62
α(A+S+A:S+R)ϕ(G+C+S+B)γ(G+B)w(G+C+S+B)g(G+B)	2444.98	2.85	0.02	63
α(S+R)ϕ(G+C+S+B+R2)γ(C)w(G+C+S+B+R2)g(C)	2445.08	2.95	0.01	59
...	...	...	...	...
$\alpha(.)\sigma(.)\phi(.)\gamma''(.)\gamma'(.)r(.)w(.)g(.)$	2758.61	316.47	0	25

Table D1. AICc results for selected ziPNE models of three New Zealand Robin populations in Fiordland National Park. Covariates included age class (A), breeding season (B), age class at time of capture (C), study area (G), sex (S), and time (T), as well as intercept only (.) and interactions (:). Three covariate models for rat control were examined, including an immediate and constant effect during the entire program (R) and no immediate effect with a delayed effect thereafter (R2). Model-specific covariates were included for log-scale mean sighting rate (α), apparent survival (ϕ), transition rates under completely random temporary emigration (γ), mark identification probability (r), and zero-inflation parameters (w and g). Unless otherwise noted, models include separate unmarked population size (U) estimates for each sighting occasion of each study area, the "across" heterogeneity model σ(S), marked identification probability model r(G+T+G:T), w_1 effects for Knobs Flat and Walker Creek study areas, and w_2 effects for the Smithy study area. The number of estimated parameters (K) includes all covariate and intercept terms. In addition to the null Markovian emigration model, only models with ΔAICc < 3 are presented.

Web Appendix E: Simulation study examining individual heterogeneity model mis-specification

We performed an additional series of simulation experiments to assess the robustness of the "within" and "across" heterogeneity models when data are generated as "across" and "within", respectively. Each simulated scenario included $T = 7$, $\phi_t = 0.95$, $\gamma''_t = \gamma'_t = 0.1$, $w_t = 0.95$, $g_t = 0.9$, $r_t = 1$, $\mathrm{E}(R_1) \in \{60, 120\}$, $\mathrm{E}(U_1) \in \{300, 600\}$, $\lambda_t \in \{1.5, 5, 100\}$, and $\tau \in \{0, 0.5, 1, 5\}$, where $\mathrm{E}(x_{s,t}) = \lambda_t$ and $\mathrm{var}(x_{s,t}) = \lambda_t(1 + \tau)$, such that $\alpha_t = 2\log(\lambda_t) - \log(\lambda_t^2 + \lambda_t\tau)/2$ and $\sigma_t = \sqrt{\log(\lambda_t^2 + \lambda_t\tau) - 2\log(\lambda_t)}$ for $s \in \mathbb{N}_T$ and $t = 1, \ldots, T$. For R_t $(t = 2, \ldots, T)$, some of the U_{t-1} unmarked individuals became newly marked (with probability 0.02) in order to maintain a somewhat consistent marked population size over time. However, no additional unmarked individuals were introduced to the population after the first occasion, so N_t is expected to steadily decline through time due to mortality and permanent emigration.

For each combination, we simulated data from both the "within" and "across" heterogeneity models, thus yielding $2 \times 2 \times 2 \times 3 \times 4 = 96$ simulated scenarios. We fit four models to each of 400 simulated data sets: 1) ziPNE within heterogeneity (Equation 3); 2) ziPNE across heterogeneity (Equation 4); 3) ztPNE within heterogeneity; and 4) ztPNE across heterogeneity. We assumed n_1 was known and n_t $(t = 2, \ldots, T)$ was unknown. For scenarios with $\tau = 0$, we fixed $\sigma_t = 0$ for each of the fitted models. For simplicity, we also fixed $r_t = 1$, $w_1 = g_1 = 1$ (because n_1 is known), and otherwise assumed no time-dependence in parameters (e.g. $\phi_1 = \phi_2 = \cdots = \phi_{T-1}$). All simulations used $L = 101$ quadrature points for numerical integration. We eval-

uated estimator performance based on bias, 95% confidence interval coverage of the true parameter values, and root mean squared error (RMSE). Simulation results are presented using the nested loop plot of Rücker and Schwarzer (2014).

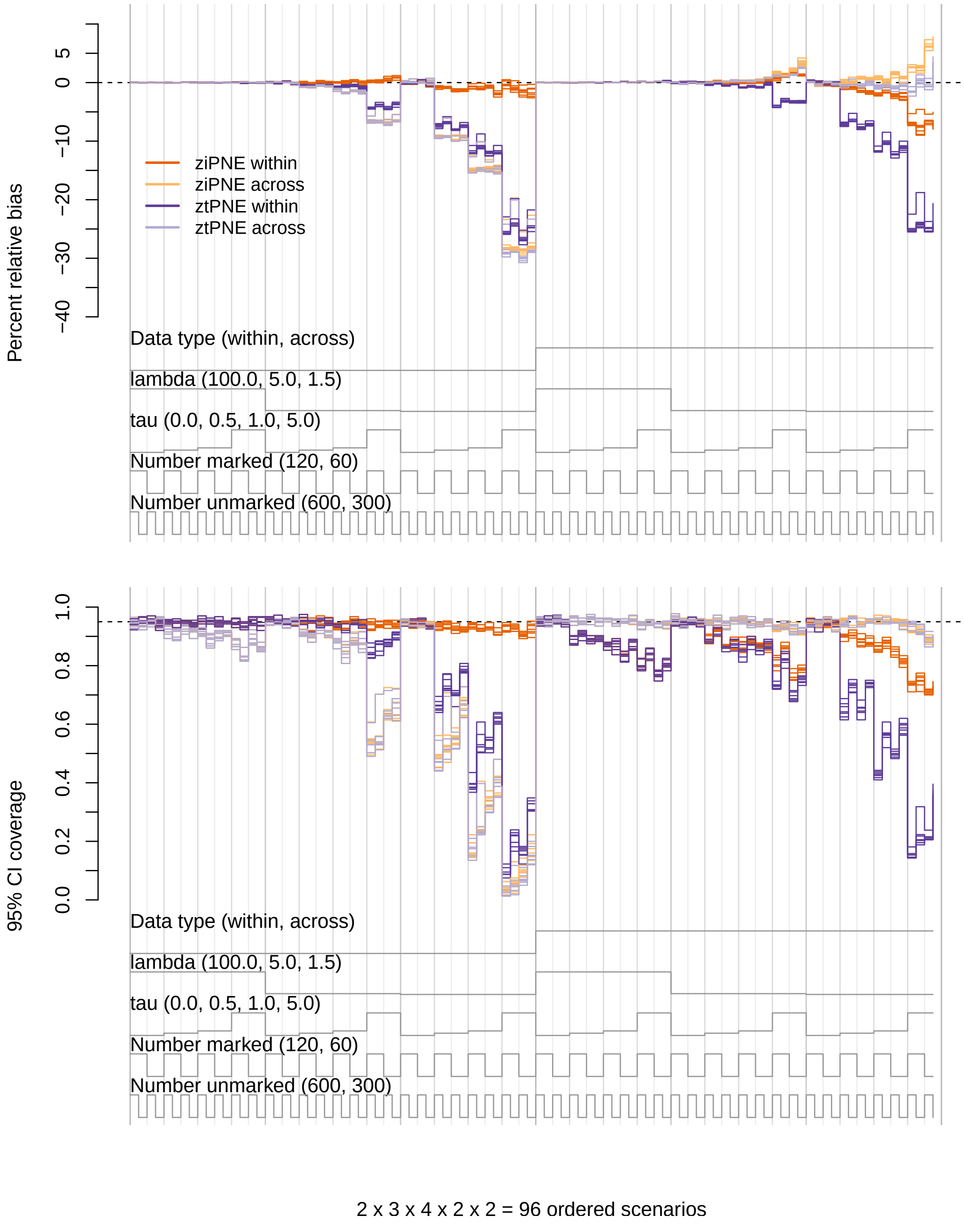

Figure E1. Nested loop plots of percent relative bias (top panel) and 95% confidence interval coverage (bottom panel) of abundance $(N_t, t = 1, \ldots, 7)$ estimators based on 96 simulated scenarios. Scenarios are ordered from outer to inner loops by the generated data type ("within" or "across" heterogeneity), $\lambda_t \in \{100, 5, 1.5\}$ ("lambda"), $\tau \in \{0, 0.5, 1, 5\}$ ("tau"), $\mathrm{E}(R_1) \in \{120, 60\}$ ("Number marked"), and $\mathrm{E}(U_1) \in \{600, 300\}$ ("Number unmarked"). Estimators include the zero-inflated Poisson log-normal estmator (*dark orange* = ziPNE within, *light orange* = ziPNE across) and the zero-truncated Poisson log-normal estimator (*dark purple* = ztPNE within, *light purple* = ztPNE across). Results are based on 400 simulated data sets

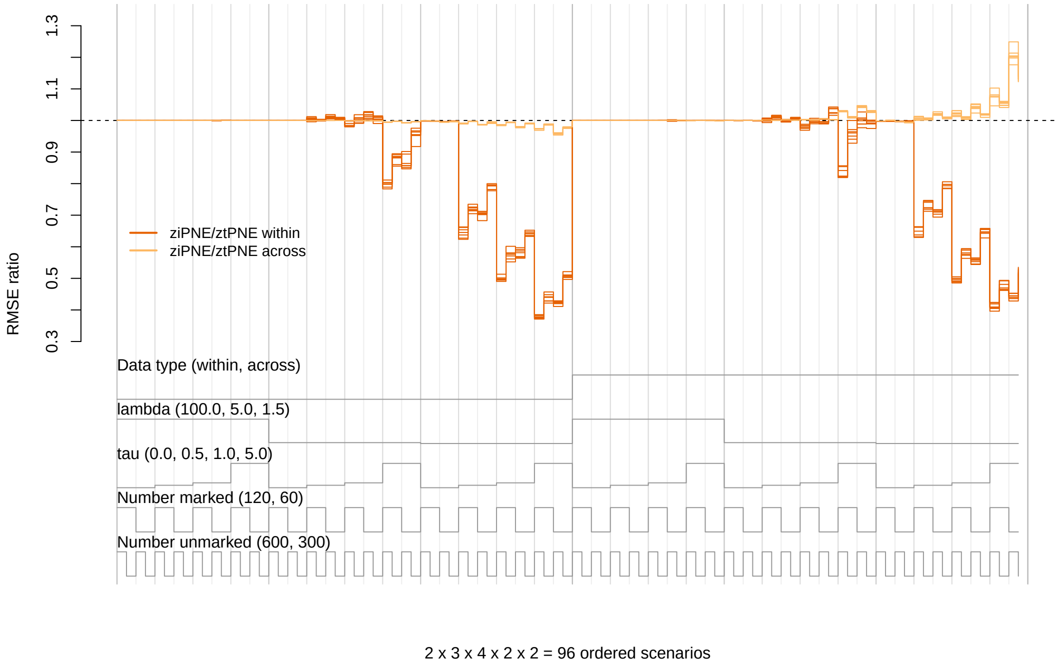

Figure E2. Nested loop plots of ratio of root mean squared errors (RMSE ratio) of the zero-inflated (ziPNE) and zero-truncated (ztPNE) Poisson log-normal abundance ($N_t, t = 1, \ldots, 7$) estimators based on 96 simulated scenarios. Scenarios are ordered from outer to inner loops by the generated data type ("within" or "across" heterogeneity), $\lambda_t \in \{100, 5, 1.5\}$ ("lambda"), $\tau \in \{0, 0.5, 1, 5\}$ ("tau"), $\mathrm{E}(R_1) \in \{120, 60\}$ ("Number marked"), and $\mathrm{E}(U_1) \in \{600, 300\}$ ("Number unmarked"). RMSE ratios were calculated as RMSE(ziPNE)/RMSE(ztPNE) for the within heterogeneity models (*dark orange*) and the across heterogeneity models (*light orange*). Results are based on 400 simulated data sets for each scenario.

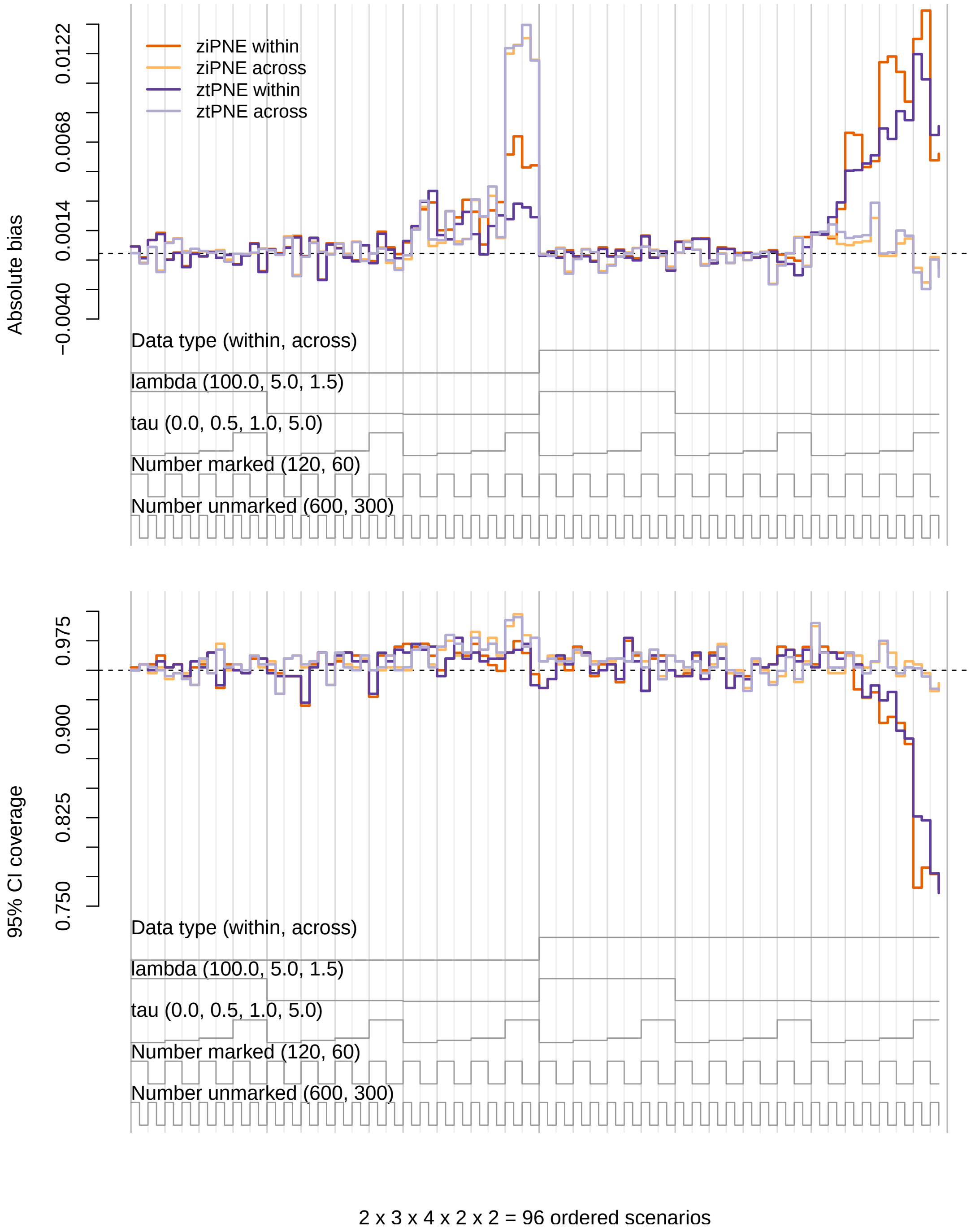

Figure E3. Nested loop plots of absolute bias (top panel) and 95% confidence interval coverage (bottom panel) of apparent survival (ϕ) estimators based on 96 simulated scenarios. Scenarios are ordered from outer to inner loops by the generated data type ("within" or "across" heterogeneity), $\lambda_t \in \{100, 5, 1.5\}$ ("lambda"), $\tau \in \{0, 0.5, 1, 5\}$ ("tau"), $\mathrm{E}(R_1) \in \{120, 60\}$ ("Number marked"), and $\mathrm{E}(U_1) \in \{600, 300\}$ ("Number unmarked"). Estimators include the zero-inflated Poisson log-normal estmator (*dark orange* = ziPNE within, *light orange* = ziPNE across) and the zero-truncated Poisson log-normal estimator (*dark purple* = ztPNE within, *light purple* = ztPNE across). Results are based on 400 simulated data sets for each scenario.

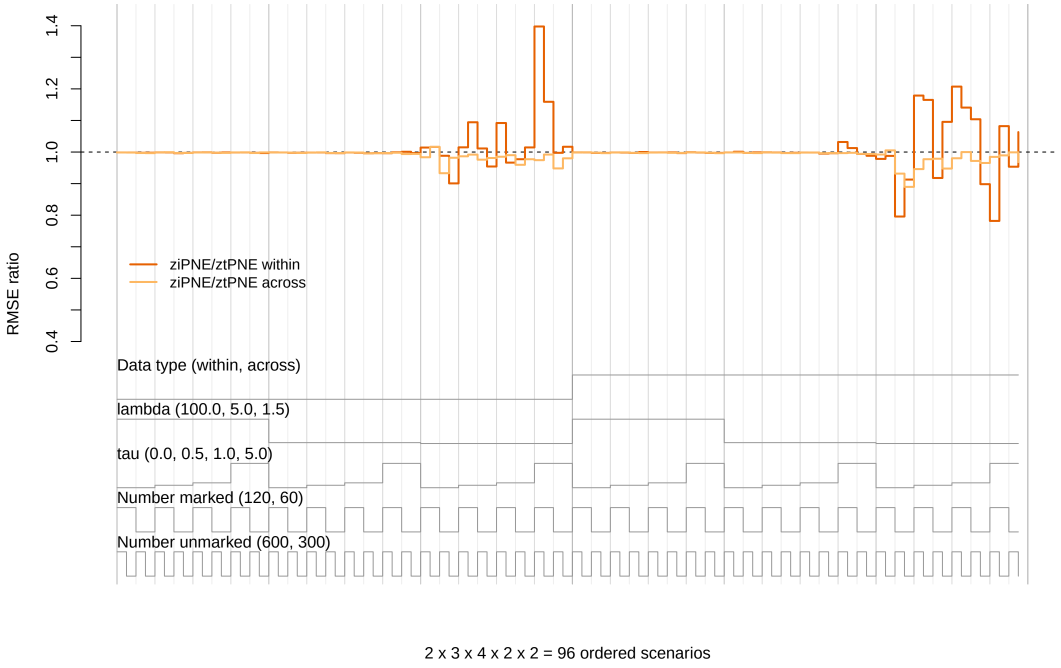

Figure E4. Nested loop plots of ratio of root mean squared errors (RMSE ratio) of the zero-inflated (ziPNE) and zero-truncated (ztPNE) Poisson log-normal apparent survival (ϕ) estimators based on 96 simulated scenarios. Scenarios are ordered from outer to inner loops by the generated data type ("within" or "across" heterogeneity), $\lambda_t \in \{100, 5, 1.5\}$ ("lambda"), $\tau \in \{0, 0.5, 1, 5\}$ ("tau"), $\mathrm{E}\,(R_1) \in \{120, 60\}$ ("Number marked"), and $\mathrm{E}\,(U_1) \in \{600, 300\}$ ("Number unmarked"). RMSE ratios were calculated as RMSE(ziPNE)/RMSE(ztPNE) for the within heterogeneity models (*dark orange*) and the across heterogeneity models (*light orange*). Results are based on 400 simulated data sets for each scenario.

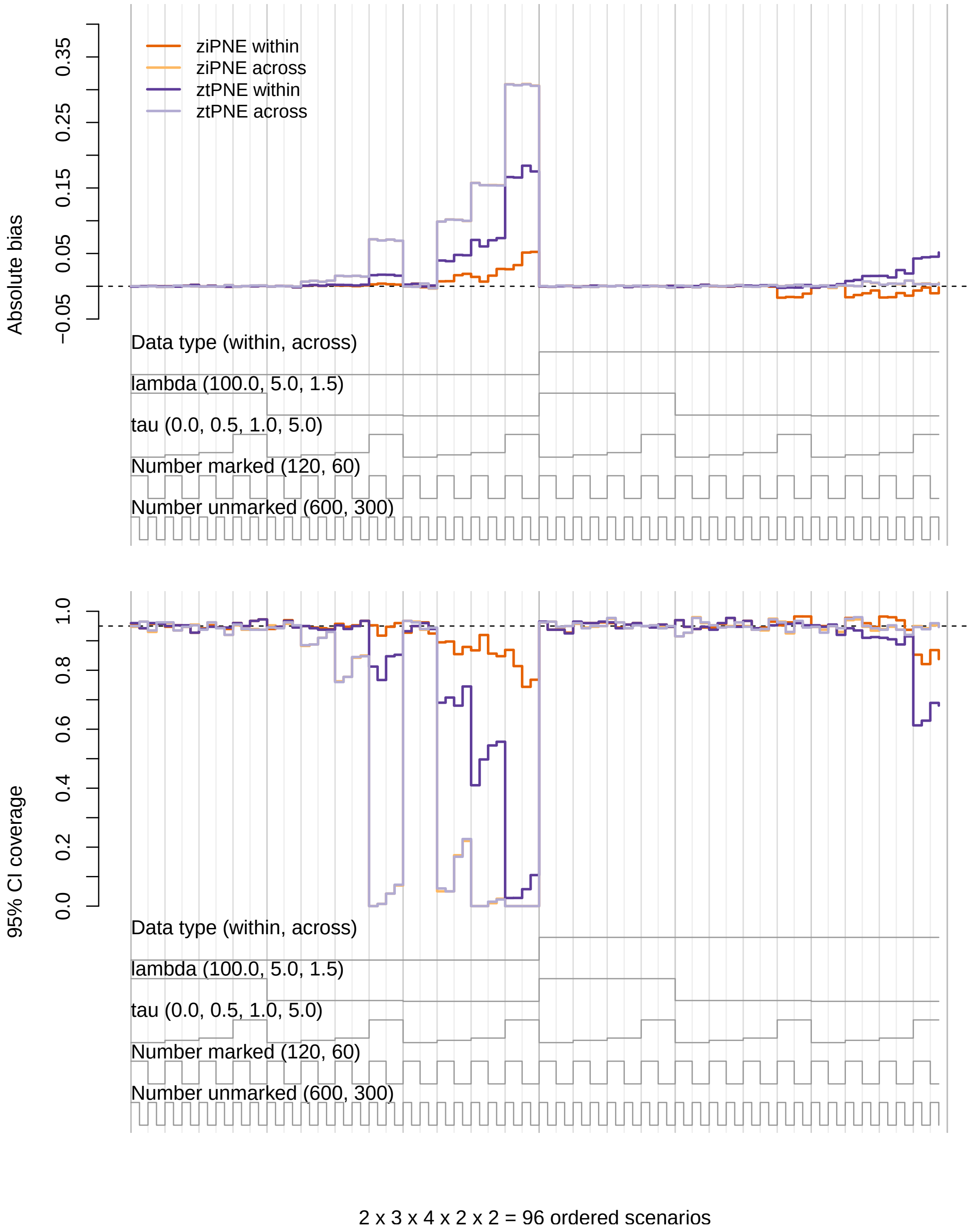

Figure E5. Nested loop plots of absolute bias (top panel) and 95% confidence interval coverage (bottom panel) of γ'' estimators based on 96 simulated scenarios. Scenarios are ordered from outer to inner loops by the generated data type ("within" or "across" heterogeneity), $\lambda_t \in \{100, 5, 1.5\}$ ("lambda"), $\tau \in \{0, 0.5, 1, 5\}$ ("tau"), $\mathrm{E}(R_1) \in \{120, 60\}$ ("Number marked"), and $\mathrm{E}(U_1) \in \{600, 300\}$ ("Number unmarked"). Estimators include the zero-inflated Poisson log-normal estmator (*dark orange* = ziPNE within, *light orange* = ziPNE across) and the zero-truncated Poisson log-normal estimator (*dark purple* = ztPNE within, *light purple* = ztPNE across). Results are based on 400 simulated data sets for each scenario.

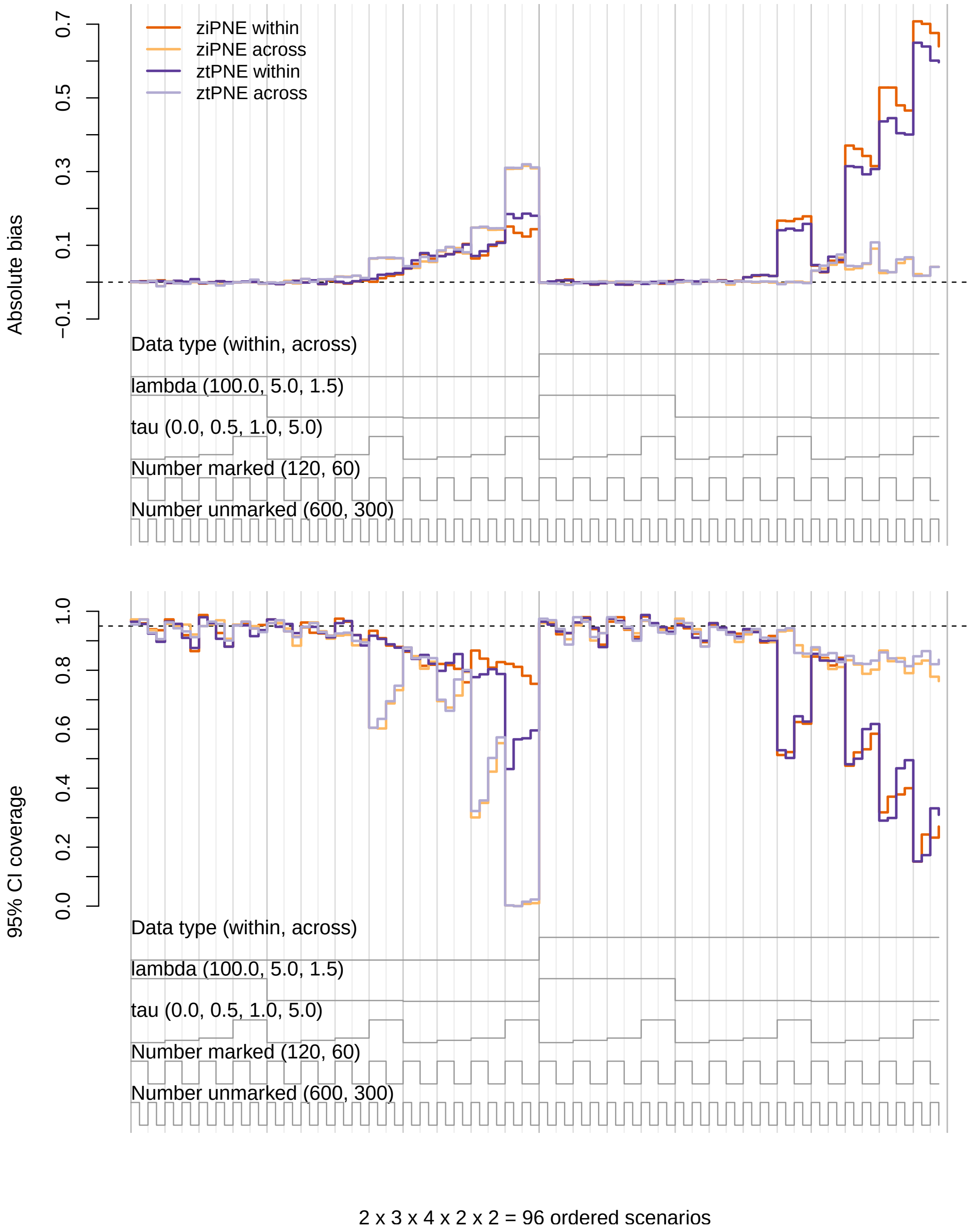

Figure E6. Nested loop plots of absolute bias (top panel) and 95% confidence interval coverage (bottom panel) of γ' estimators based on 96 simulated scenarios. Scenarios are ordered from outer to inner loops by the generated data type ("within" or "across" heterogeneity), $\lambda_t \in \{100, 5, 1.5\}$ ("lambda"), $\tau \in \{0, 0.5, 1, 5\}$ ("tau"), $\mathrm{E}(R_1) \in \{120, 60\}$ ("Number marked"), and $\mathrm{E}(U_1) \in \{600, 300\}$ ("Number unmarked"). Estimators include the zero-inflated Poisson log-normal estmator (*dark orange* = ziPNE within, *light orange* = ziPNE across) and the zero-truncated Poisson log-normal estimator (*dark purple* = ztPNE within, *light purple* = ztPNE across). Results are based on 400 simulated data sets for each scenario.

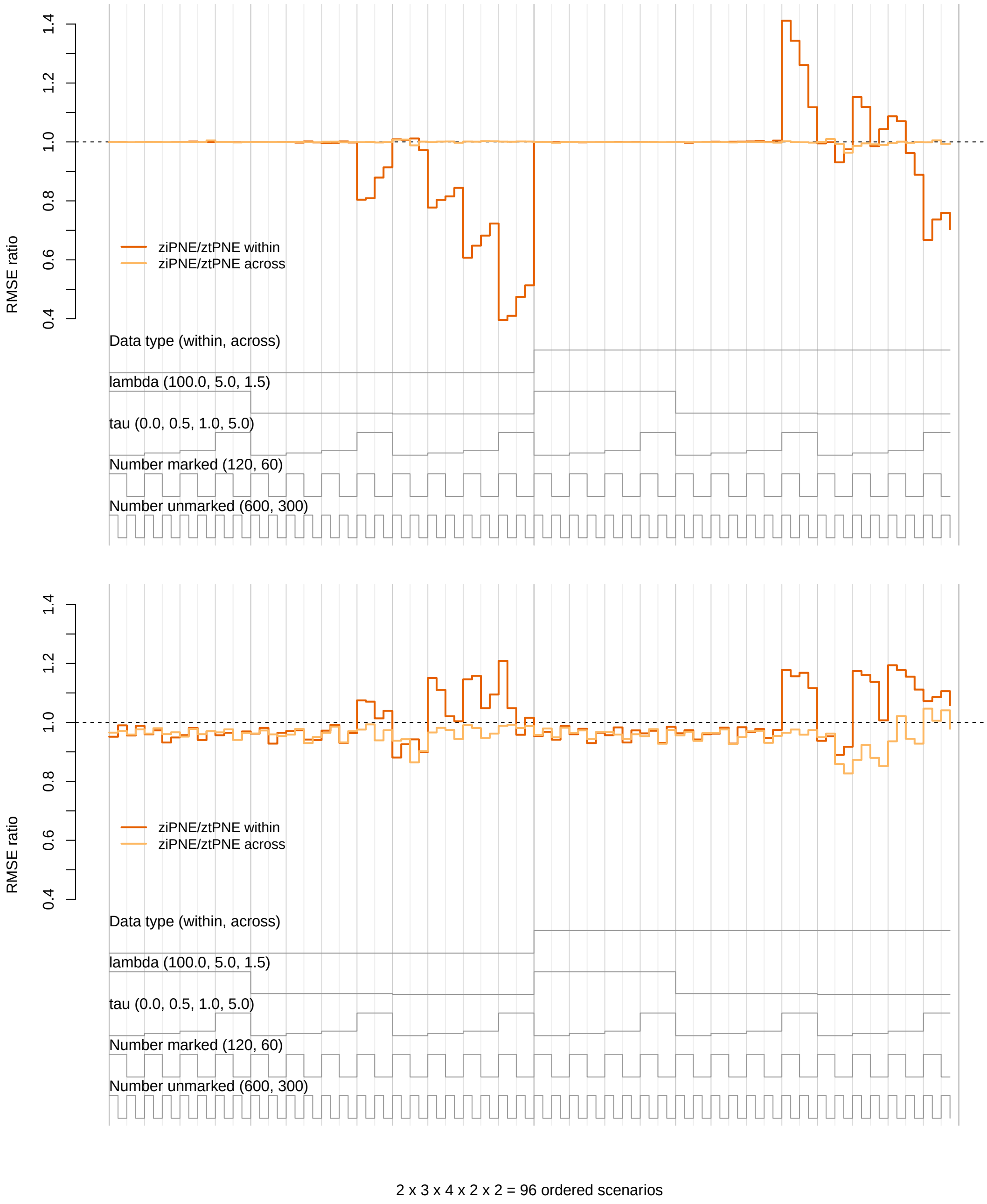

Figure E7. Nested loop plots of ratio of root mean squared errors (RMSE ratio) of the zero-inflated (ziPNE) and zero-truncated (ztPNE) Poisson log-normal γ'' (top panel) and γ' (lower panel) estimators based on 96 simulated scenarios. Scenarios are ordered from outer to inner loops by the generated data type ("within" or "across" heterogeneity), $\lambda_t \in \{100, 5, 1.5\}$ ("lambda"), $\tau \in \{0, 0.5, 1, 5\}$ ("tau"), $\mathrm{E}(R_1) \in \{120, 60\}$ ("Number marked"), and $\mathrm{E}(U_1) \in \{600, 300\}$ ("Number unmarked"). RMSE ratios were calculated as RMSE(ziPNE)/RMSE(ztPNE) for the within heterogeneity models (*dark orange*) and the across heterogeneity models (*light orange*). Results are based on 400 simulated data sets for each scenario.

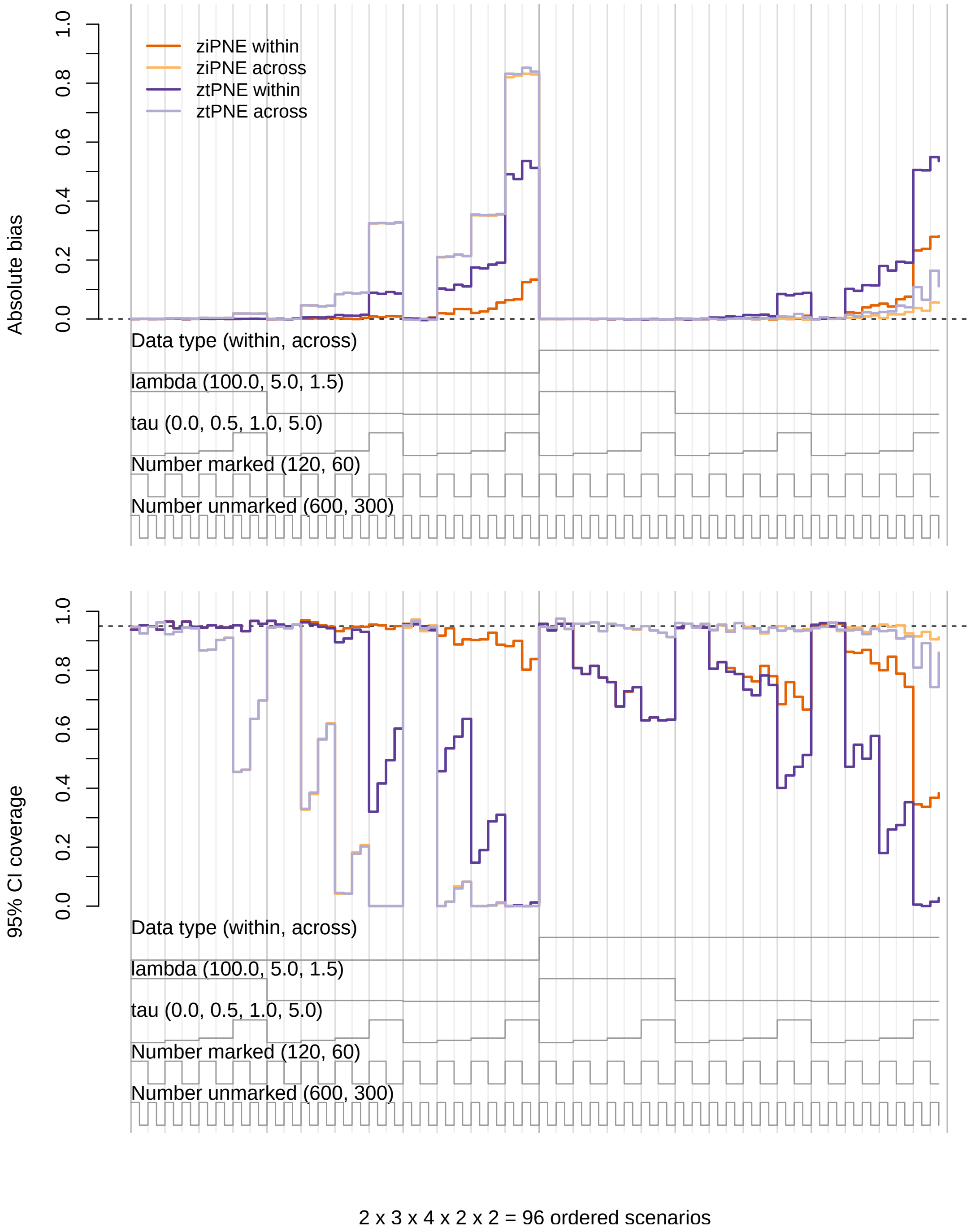

Figure E8. Nested loop plots of absolute bias (top panel) and 95% confidence interval coverage (bottom panel) of α estimators based on 96 simulated scenarios. Scenarios are ordered from outer to inner loops by the generated data type ("within" or "across" heterogeneity), $\lambda_t \in \{100, 5, 1.5\}$ ("lambda"), $\tau \in \{0, 0.5, 1, 5\}$ ("tau"), $\mathrm{E}(R_1) \in \{120, 60\}$ ("Number marked"), and $\mathrm{E}(U_1) \in \{600, 300\}$ ("Number unmarked"). Estimators include the zero-inflated Poisson log-normal estmator (*dark orange* = ziPNE within, *light orange* = ziPNE across) and the zero-truncated Poisson log-normal estimator (*dark purple* = ztPNE within, *light purple* = ztPNE across). Results are based on 400 simulated data sets for each scenario.

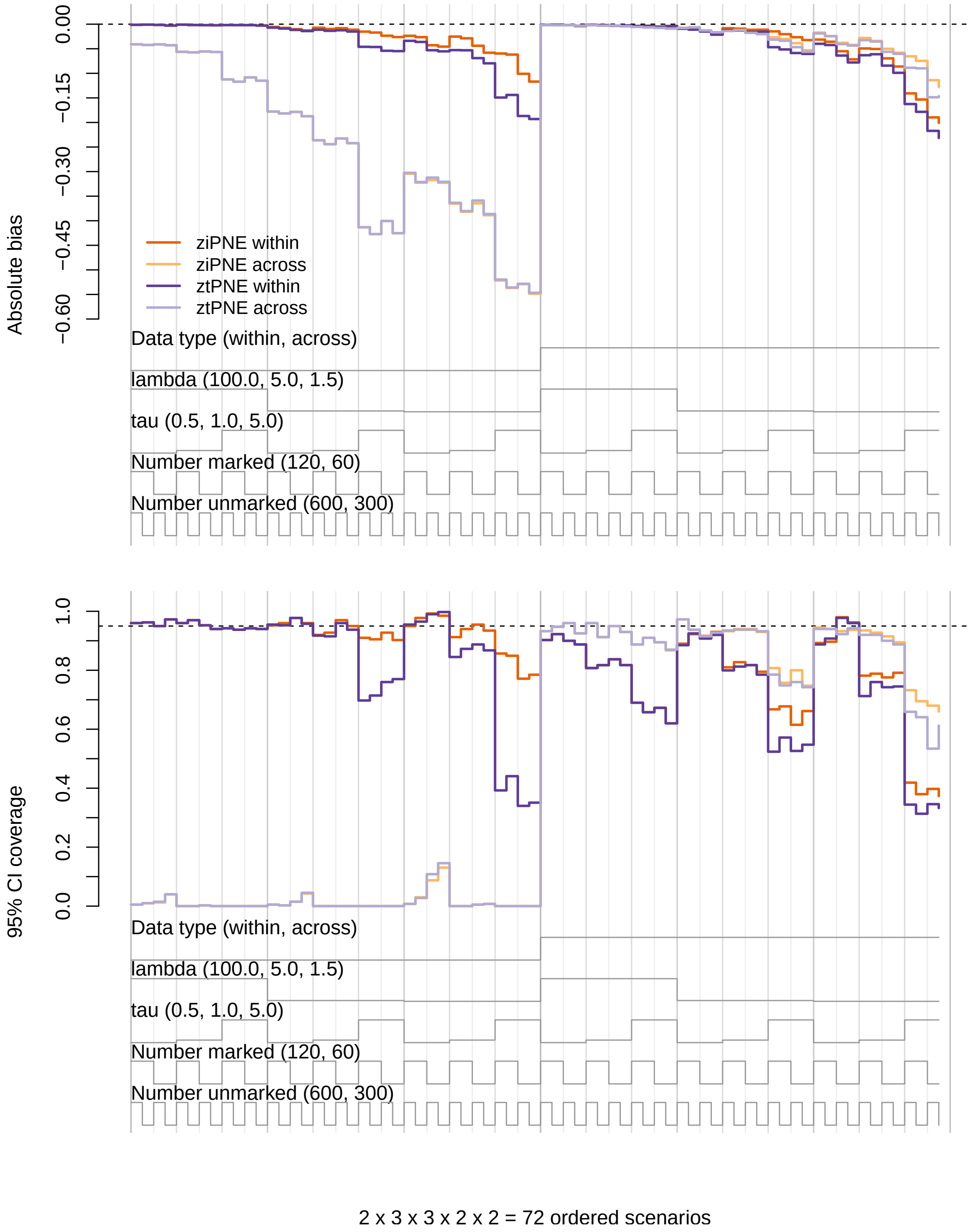

Figure E9. Nested loop plots of absolute bias (top panel) and 95% confidence interval coverage (bottom panel) of σ estimators based on 72 simulated scenarios. Scenarios are ordered from outer to inner loops by the generated data type ("within" or "across" heterogeneity), $\lambda_t \in \{100, 5, 1.5\}$ ("lambda"), $\tau \in \{0.5, 1, 5\}$ ("tau"), $\mathrm{E}(R_1) \in \{120, 60\}$ ("Number marked"), and $\mathrm{E}(U_1) \in \{600, 300\}$ ("Number unmarked"). Estimators include the zero-inflated Poisson log-normal estmator (*dark orange* = ziPNE within, *light orange* = ziPNE across) and the zero-truncated Poisson log-normal estimator (*dark purple* = ztPNE within, *light purple* = ztPNE across). Results are based on 400 simulated data sets for each scenario.

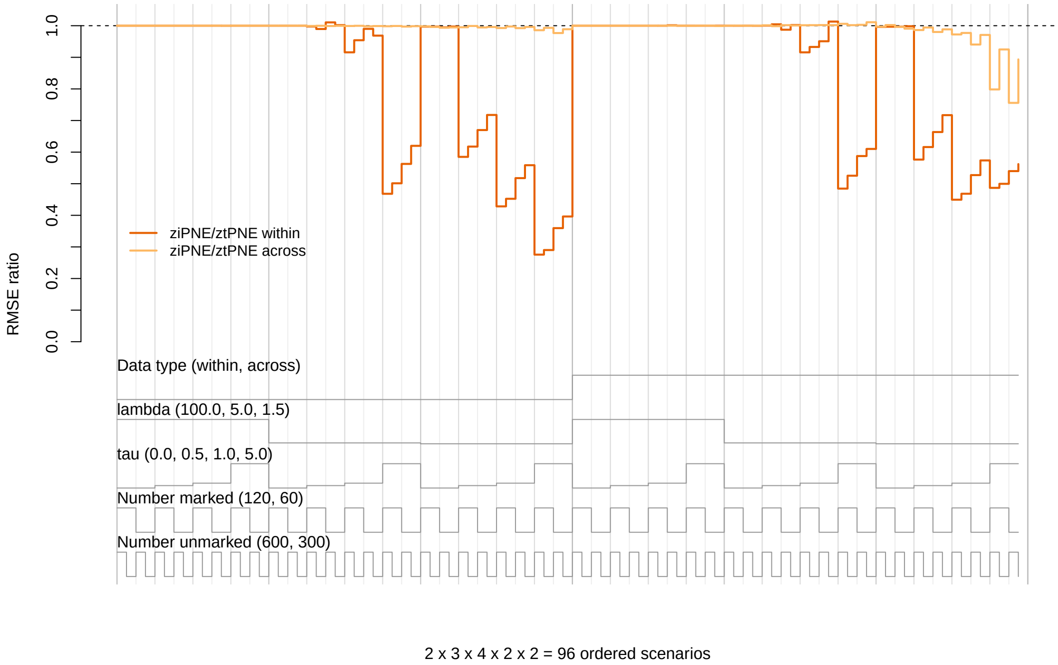

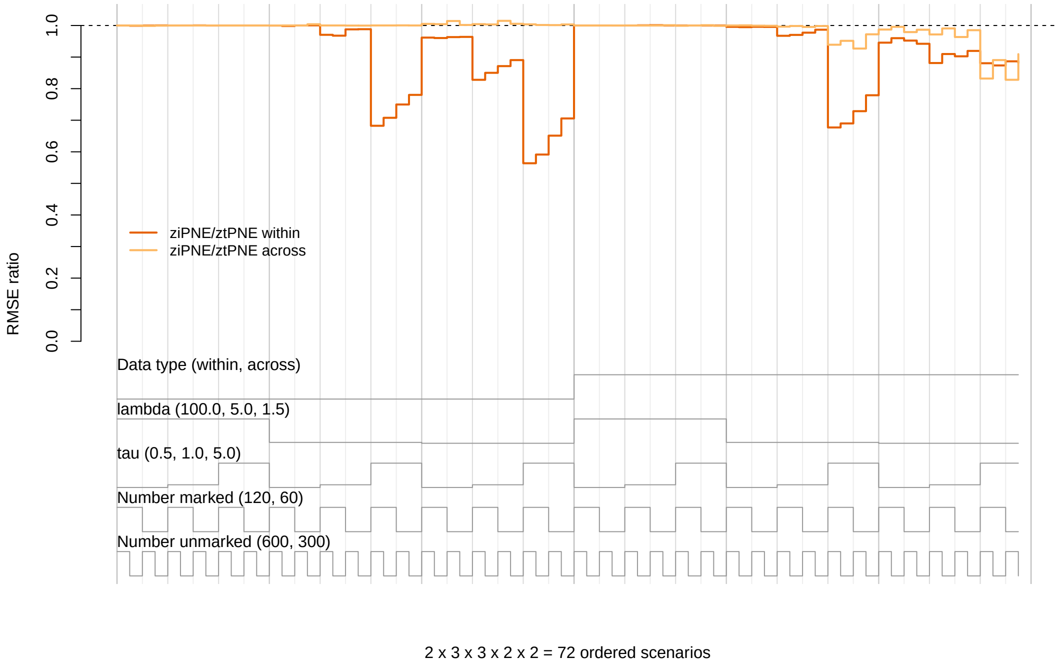

Figure E10. Nested loop plots of ratio of root mean squared errors (RMSE ratio) of the zero-inflated (ziPNE) and zero-truncated (ztPNE) Poisson log-normal α (top panel) and σ (lower panel) estimators based on simulated scenarios. Scenarios are ordered from outer to inner loops by the generated data type ("within" or "across" heterogeneity), $\lambda_t \in \{100, 5, 1.5\}$ ("lambda"), $\tau \in \{0, 0.5, 1, 5\}$ ("tau"), $\mathrm{E}(R_1) \in \{120, 60\}$ ("Number marked"), and $\mathrm{E}(U_1) \in \{600, 300\}$ ("Number unmarked"). RMSE ratios were calculated as RMSE(ziPNE)/RMSE(ztPNE) for the within heterogeneity models (*dark orange*) and the across heterogeneity models (*light orange*). Results are based on 400 simulated data sets for each scenario.

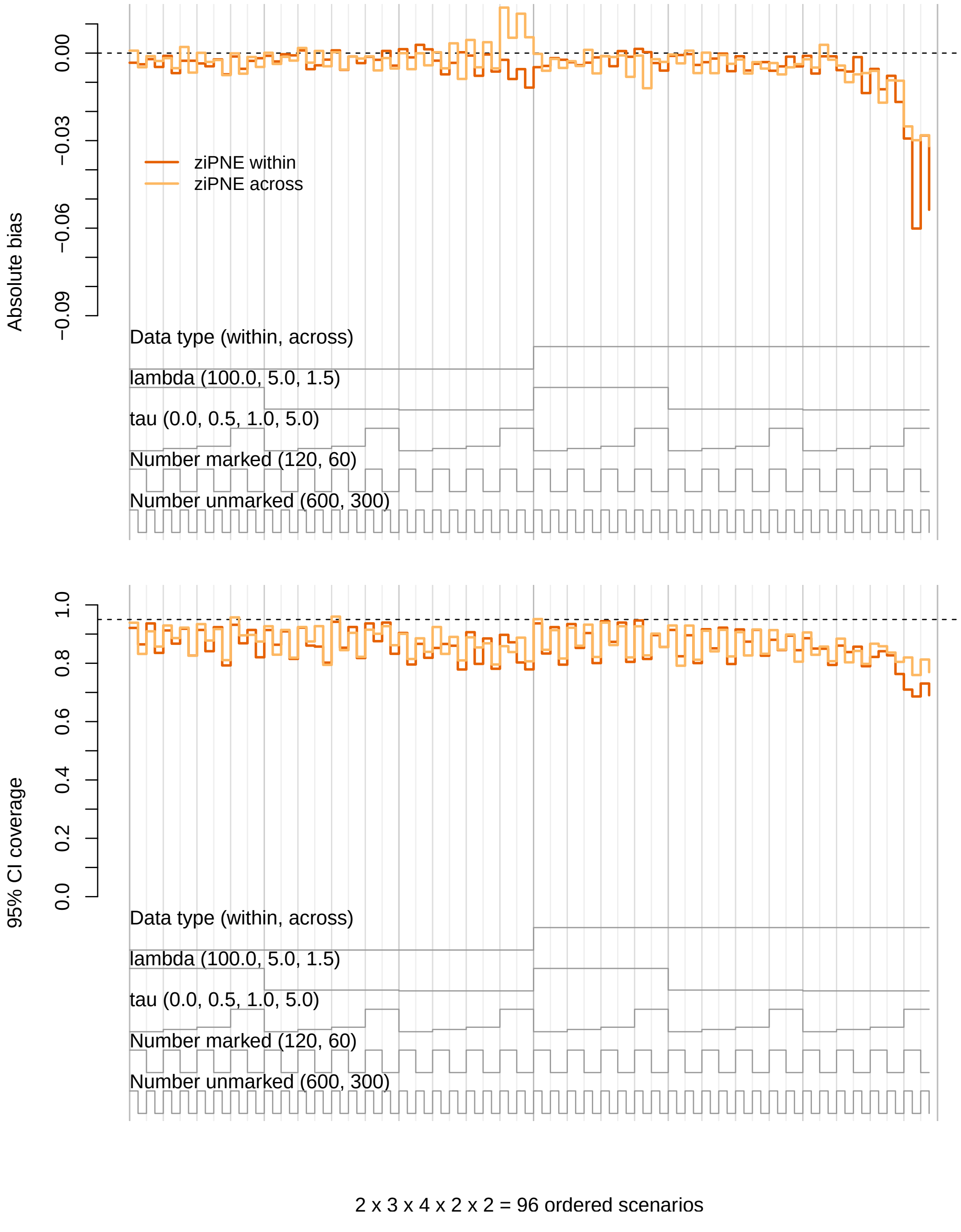

Figure E11. Nested loop plots of absolute bias (top panel) and 95% confidence interval coverage (bottom panel) of w estimators based on 96 simulated scenarios. Scenarios are ordered from outer to inner loops by the generated data type ("within" or "across" heterogeneity), $\lambda_t \in \{100, 5, 1.5\}$ ("lambda"), $\tau \in \{0, 0.5, 1, 5\}$ ("tau"), $\mathrm{E}(R_1) \in \{120, 60\}$ ("Number marked"), and $\mathrm{E}(U_1) \in \{600, 300\}$ ("Number unmarked"). Estimators include the zero-inflated Poisson log-normal estmator with "within" heterogeneity (*dark orange*) and "across" heterogeneity (*light orange*). Results are based on 400 simulated data sets for each scenario.

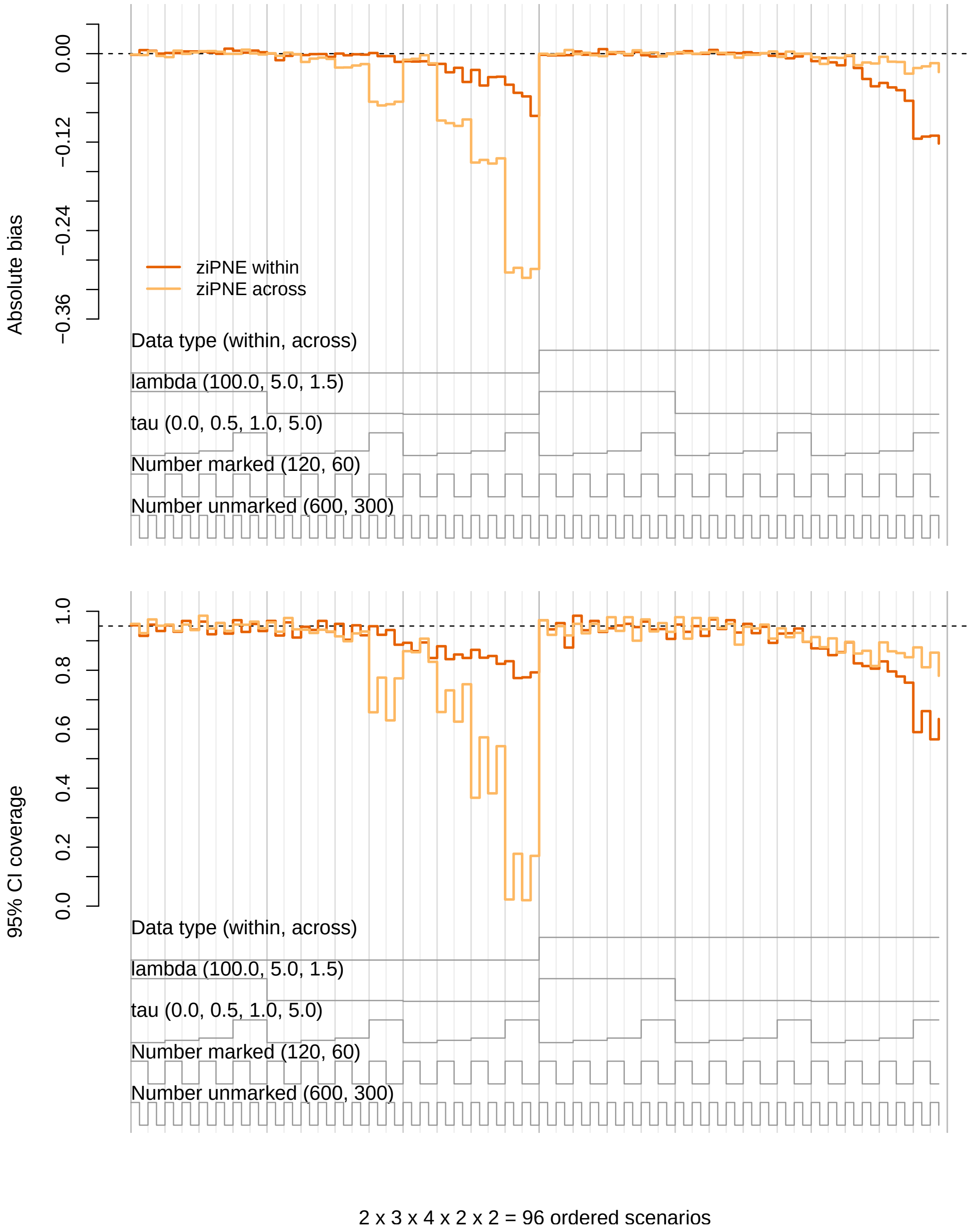

Figure E12. Nested loop plots of absolute bias (top panel) and 95% confidence interval coverage (bottom panel) of g estimators based on 96 simulated scenarios. Scenarios are ordered from outer to inner loops by the generated data type ("within" or "across" heterogeneity), $\lambda_t \in \{100, 5, 1.5\}$ ("lambda"), $\tau \in \{0, 0.5, 1, 5\}$ ("tau"), $\mathrm{E}(R_1) \in \{120, 60\}$ ("Number marked"), and $\mathrm{E}(U_1) \in \{600, 300\}$ ("Number unmarked"). Estimators include the zero-inflated Poisson log-normal estmator with "within" heterogeneity (*dark orange*) and "across" heterogeneity (*light orange*). Results are based on 400 simulated data sets for each scenario.

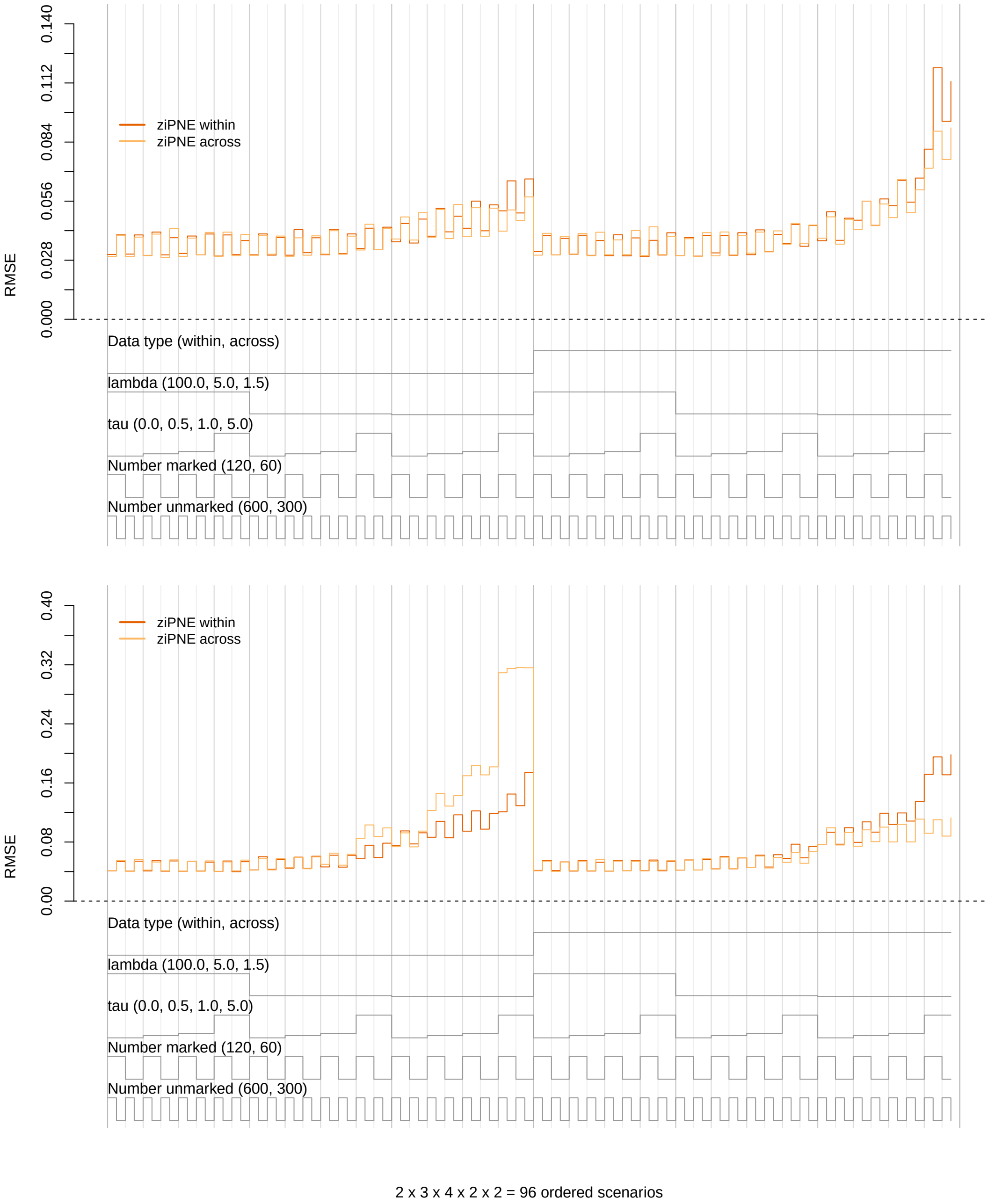

Figure E13. Root mean squared error (RMSE) of w (top panel) and g (lower panel) estimators based on 96 simulated scenarios. Scenarios are ordered from outer to inner loops by the generated data type ("within" or "across" heterogeneity), $\lambda_t \in \{100, 5, 1.5\}$ ("lambda"), $\tau \in \{0, 0.5, 1, 5\}$ ("tau"), $\mathrm{E}(R_1) \in \{120, 60\}$ ("Number marked"), and $\mathrm{E}(U_1) \in \{600, 300\}$ ("Number unmarked"). Estimators include the zero-inflated Poisson log-normal estmator with "within" heterogeneity (*dark orange*) and "across" heterogeneity (*light orange*). Results are based on 400 simulated data sets for each scenario.
