# Supplementary material for: Improved methods for estimating abundance and related demographic parameters from mark‐resight data
Source: Biometrics. 2019 Apr 25;75(3):799–809. doi: 10.1111/biom.13058 (PMC6850357; doi:10.1111/biom.13058)
Supplement: Supplementary file 3 — Supplementary Information [file BIOM-75-799-s003.pdf]

6 Supporting Information

Additional supporting information may be found online in the Supporting Information section at the end of the article. This includes Web Appendices, Tables, Figures, and code referenced in Sections 2, 3, and 5. The New Zealand robin data and R code for the analysis in Section 4 are also available. Program MARK is freely available at `http://www.phidot.org/software/mark/downloads/`. The RMark package for R is hosted on CRAN at `https://CRAN.R-project.org/package=RMark`.
